# Fecal microbiota transplantation alleviates DSS-induced colitis: increased fecal butyrate, reduced colonic p65 phosphorylation, and altered Th17/Treg ratios in the spleen and mesenteric lymph nodes

**DOI:** 10.3389/fimmu.2026.1903198

**Published:** 2026-08-17

**Authors:** Qinghua Luo, Yifan Ding, Pan Shen, Ting Chen, Zhiqiang Xu, Leichang Zhang

**Affiliations:** 1 Department of Anorectal Surgery, Affiliated Hospital of Jiangxi University of Chinese Medicine, Nanchang, China; 2 Clinical Medical College, Jiangxi University of Chinese Medicine, Nanchang, China; 3 Formula-Pattern Research Center, Jiangxi University of Chinese Medicine, Jiangxi, China

**Keywords:** butyrate, fecal microbiota transplantation, gut microbiota, p65 phosphorylation, Th17/Treg proportions

## Abstract

**Background:**

Ulcerative colitis (UC) development and progression are associated with intestinal dysbiosis, altered short-chain fatty acid (SCFA) metabolism, and immune dysregulation. Although fecal microbiota transplantation (FMT) has therapeutic potential, its key effector metabolites and regulatory pathways remain unclear.

**Methods:**

Male BALB/c mice with 2.5% dextran sulfate sodium (DSS)-induced colitis received FMT, 5-aminosalicylic acid (5-ASA), sodium butyrate (NaB), or pyrrolidine dithiocarbamate (PDTC). Colitis severity was evaluated using body weight, disease activity index, colon length, and hematoxylin and eosin staining. Cytokines in colonic tissue and serum were quantified by enzyme-linked immunosorbent assay. Colonic p-p65/p65 and p-IκBα/IκBα ratios and tight-junction proteins were measured by Western blotting. Th17/Treg proportions in the spleen and mesenteric lymph nodes were assessed by flow cytometry. Gut microbiota composition and fecal SCFA profiles were examined by 16S rRNA sequencing and targeted metabolomics, respectively.

**Results:**

Compared with DSS, FMT markedly attenuated weight loss, disease activity, colon shortening, and histopathological injury. In colonic tissue, FMT reduced IL-17A and IL-21 and increased TGF-β1. In serum, it increased IL-10 and TGF-β1 and reduced IL-21. FMT increased colonic Claudin-1, Occludin, and zonula occludens-1 expression and reduced the colonic p-p65/p65 ratio. It also reduced Th17 proportions and increased Treg proportions in the spleen and mesenteric lymph nodes. Microbiota analysis indicated improved diversity and reshaping of DSS-induced dysbiosis, including recovery of Firmicutes at the phylum level and Lachnospiraceae at the family level. FMT was associated with higher endpoint fecal acetate and butyrate concentrations than DSS, with butyrate also higher than in the 5-ASA group, supporting its evaluation as an FMT-associated readout. In a separate experiment, NaB, PDTC, and combined NaB/PDTC treatments were each associated with changes in selected outcomes relative to DSS.

**Conclusion:**

FMT alleviates DSS-induced colitis and is associated with microbiota shifts, increased endpoint fecal butyrate concentration, and a reduced colonic p-p65/p65 ratio. It is also associated with increased colonic tight-junction protein expression and altered Th17/Treg proportions in the spleen and mesenteric lymph nodes. These findings represent parallel associations and do not establish causal relationships among the measured outcomes.

## 1 Introduction

Ulcerative colitis (UC) represents a chronic, relapsing inflammatory bowel disease that predominantly involves the colonic mucosa and submucosa. It usually begins in the rectum and extends proximally, with severe cases potentially affecting the entire colon (1). Its major pathological characteristics include crypt abscess formation, goblet cell depletion, and mucosal ulceration (2). In recent years, the burden of UC has steadily increased. Although UC was historically more common in industrialized areas, including North America and Western Europe, its incidence has risen substantially in developing regions such as Asia, Africa, and South America, in parallel with lifestyle westernization. Therefore, UC has increasingly become a global public health concern (3, 4). Gut microbiota dysbiosis, altered microbial metabolism, and disrupted intestinal immune homeostasis are now widely considered key mechanisms driving UC onset and progression. Among UC-associated pathological alterations, short-chain fatty acid (SCFA) metabolic imbalance is especially evident. SCFAs, mainly acetate, propionate, and butyrate, are generated via gut microbiota-mediated fermentation of dietary fiber (5). Among microbiota-derived short-chain fatty acids, butyrate contributes to epithelial barrier integrity and mucosal repair, linking microbial metabolism to host epithelial homeostasis (6). In ulcerative colitis, inflamed mucosa displays reduced butyrate-producing capacity, suggesting that dysbiosis-associated reductions in butyrate availability may contribute to impaired epithelial barrier homeostasis (7). The NF-κB signaling pathway exhibits significant regulatory function in this process. As a key transcription factor controlling inflammatory and immune responses, NF-κB is activated by pro-inflammatory stimuli and subsequently translocates into the nucleus, where it induces inflammation-related gene transcription (8–10). During chronic inflammation, persistent NF-κB activation may further intensify SCFA metabolic dysregulation, forming a self-amplifying pathological cycle. Additional studies have shown that aberrant NF-κB activation promotes Th17 cell differentiation and inhibits regulatory T (Treg) cell function, thereby disrupting the Th17/Treg balance. SCFAs can inhibit NF-κB signaling activation, promote Treg cell differentiation, and suppress Th17 cell polarization, thus regulating intestinal inflammatory responses (11–14).

Given the central role of the gut microbiota-metabolite-immune network in UC pathogenesis, fecal microbiota transplantation (FMT), an important strategy for restoring intestinal microecology, has attracted considerable attention in UC treatment in recent years. Existing evidence indicates that FMT can increase gut microbiota diversity, modulate microbial community composition, and improve intestinal mucosal barrier function and inflammatory status. Notably, in patients who respond to FMT treatment, increased *Faecalibacterium* abundance is closely associated with higher Foxp3 expression, lower RORγt expression, and correction of Th17/Treg imbalance, suggesting that FMT may exert therapeutic effects by modulating bacterial composition and the immune microenvironment (12). Meanwhile, animal studies have demonstrated that FMT reduces inflammation-related signaling molecules, including NF-κB p65, STAT3, and IL-6, thereby mitigating colonic inflammatory injury (13). Recent systematic reviews and meta-analyses further indicate that FMT provides certain benefits in inducing clinical and endoscopic remission in UC. However, its efficacy is still affected by multiple factors, including donor screening, bacterial suspension preparation, administration route, intervention frequency, and recipient baseline microbiota status. Notably, higher microbiota diversity and an increased relative abundance of Lachnospiraceae at the family level may be associated with the therapeutic effects of FMT (14). Moreover, the latest clinical guidelines and consensus statements suggest that the clinical potential of FMT in UC warrants attention, although optimal implementation strategies and mechanisms of action require further clarification (15).

Overall, current evidence suggests that FMT may alleviate UC by altering gut microbial composition, fecal SCFA profiles, and immune-associated measures. This study therefore examined whether FMT-related disease improvement was accompanied by changes in endpoint fecal butyrate concentration, the colonic p-p65/p65 and p-IκBα/IκBα ratios, and Th17/Treg proportions in the spleen and mesenteric lymph nodes. NaB and PDTC interventions were evaluated in a separate experiment to determine whether selected measured outcomes changed relative to DSS. The analyses were designed to assess parallel associations rather than a causal mechanistic network.

## 2 Materials and methods

### 2.1 Drug

Mesalazine (5-ASA, MW = 153.14) was acquired from Sigma-Aldrich (Shanghai, China); dextran sulfate sodium (DSS, MW = 36–50 kDa) was procured from MP Biomedicals (Irvine, CA, USA); sodium butyrate (NaB, purity ≥ 98%, molecular formula C_4_H_7_NaO_2_) was obtained from Sigma-Aldrich (Shanghai, China); and pyrrolidine dithiocarbamate (PDTC, as an NF-κB inhibitor, purity ≥ 98%, molecular formula C_5_H_10_NS_2_·NH_4_) was procured from MedChemExpress (Shanghai, China).

### 2.2 Reagents

The main reagents were as follows. Mouse interleukin (IL)-10, IL-17A, IL-21, IL-22, and transforming growth factor (TGF)-β1 enzyme-linked immunosorbent assay (ELISA) kits were obtained from Ruixin. Radio immunoprecipitation assay lysis buffer was purchased from Solarbio; bicinchoninic acid (BCA) protein quantification kit from Biosharp; MOPS-SDS Running Buffer and FuturePAGE™ precast protein gels from ACE; and multicolor prestained protein marker from Yeasen. QuickBlock™ Western blocking buffer and antibody diluent were obtained from Beyotime. Flow cytometry antibodies, including phycoerythrin (PE) anti-mouse CD4, allophycocyanin (APC) anti-mouse IL-17A, PE/Cyanine7 anti-mouse CD25, and fluorescein isothiocyanate (FITC) anti-mouse/rat Foxp3, were purchased from Elabscience; CD16/CD32 antibody was procured from Cell Signaling. Phorbol 12-myristate 13-acetate (PMA), ionomycin, and Brefeldin A were procured from Solarbio; Collagenase II from Servicebio; and red blood cell lysis buffer from Meilun Biotechnology. The intracellular fixation/permeabilization kit and cell staining buffer were obtained from Elabscience. For microbiome analysis, the E.Z.N.A.^®^ Soil DNA Kit was procured from Omega Bio-Tek; FastPfu Polymerase from TransGen; AxyPrep DNA Gel Extraction Kit from Axygen Biosciences; NEXTFLEX^®^ Rapid DNA-Seq Kit from Bioo Scientific; and sequencing kits from Illumina. Acetic acid, propionic acid, butyric acid, isobutyric acid, valeric acid, isovaleric acid, caproic acid, and isocaproic acid (internal standard) for SCFAs detection were purchased from Sigma.

### 2.3 Animals

Five-week-old male SPF-grade BALB/c mice [body weight: (20 ± 2) g] were procured from GemPharmatech Co., Ltd. (Nanjing, China). All mice were maintained in a 10,000-class barrier animal facility under standardized environmental conditions, with unrestricted access to standard rodent chow and sterile drinking water. All animal procedures received review, approval, and supervision from the Institutional Animal Care and Use Committee at Jiangxi University of Chinese Medicine (Ethics Approval No.: JZLLSC20250517; Approval Date: May 17, 2025).

### 2.4 DSS-induced colitis and FMT treatment

After 3 days of acclimatization, mice were arbitrarily split into four groups (n = 10 per group): normal control (Control), model (DSS), FMT (FMT), and 5-ASA positive control (5-ASA). Apart from the normal control group that received drinking water freely, all remaining groups were given freshly prepared 2.5% DSS solution ad libitum for 7 days to develop a UC model (16). After modeling, DSS exposure was stopped in all groups. The FMT group received fecal bacterial suspension via intragastric gavage once daily for 7 consecutive days, while the 5-ASA group was given 5-ASA solution through intragastric gavage once daily for 7 consecutive days. Each administration volume was 0.2 mL. DSS was not provided during this intervention period. Thereafter, all groups were re-exposed to 2.5% DSS solution for 7 days to maintain the inflammatory model. At the experimental endpoint, mice were deeply anesthetized with isoflurane inhalation until loss of the pedal withdrawal reflex. After deep anesthesia was confirmed, mice were euthanized by cervical dislocation before sample collection, in accordance with the approved animal protocol. No injectable anesthetic was used for this terminal procedure. This procedure was selected to minimize animal pain and distress during terminal tissue harvesting. The fecal bacterial suspension used for FMT was prepared from fresh morning feces collected from mice in the normal control group. Fresh fecal samples collected from the normal control group were pooled, and a total of 4 g of pooled fecal material was used for each preparation. All subsequent processing procedures were performed under sterile anaerobic conditions in an anaerobic workstation to minimize oxygen exposure during FMT preparation. Briefly, 4 g of fresh feces was weighed, vortex-mixed with five volumes of normal saline, and sequentially filtered through stainless steel sieves with pore sizes of 2.0, 1.0, 0.5, and 0.25 mm. The filtrate underwent centrifugation at 6,000 r/min for 15 min, after which the bacterial pellet was collected, resuspended, and subjected to three washes with phosphate-buffered saline under sterile anaerobic conditions. The suspension was then subjected to three to five additional cycles of resuspension in five volumes of normal saline, followed by repeated centrifugation and washing. The final bacterial pellet was resuspended in three volumes of normal saline and preserved at −80°C until use. Recipient mice were cohoused with mice from the same experimental group throughout the intervention period, with no cross-group cohousing (14). The 5-ASA solution was prepared at 300 mg/kg by dissolving 0.3 mg of 5-ASA per gram of body weight in 0.2 mL of distilled water, with fresh solutions prepared daily before administration. On day 21, mice were deeply anesthetized with isoflurane inhalation until loss of the pedal withdrawal reflex and euthanized by cervical dislocation. Subsequently, blood samples, mesenteric lymph nodes, and colonic tissues were harvested for further analyses. For Experiment 1, three mice per group were randomly selected based on animal identification numbers. Samples from the same three mice were used for microbiome, endpoint fecal SCFA, Western blotting, immunofluorescence, and flow cytometry analyses.

### 2.5 DSS-induced colitis and NaB intervention

A separate cohort of forty-five male BALB/c mice (5 weeks old) underwent a 3-day acclimatization period before randomly allocated using into five groups of nine animals each: control (Control), model (DSS), NaB (NaB), PDTC (PDTC), and PDTC + NaB (PDTC+NaB). Apart from the control group that had free access to regular drinking water, all remaining groups received 2.5% DSS solution ad libitum for 7 days to develop a UC mouse model. After modeling, DSS exposure was stopped in all groups. NaB was administered by oral gavage at 300 mg/kg/day to the NaB and PDTC+NaB groups. The NaB dose was selected with reference to a previous DSS-induced colitis study that included oral butyrate at 300 mg/kg (17). PDTC was administered by intraperitoneal injection at 100 mg/kg to the PDTC and PDTC+NaB groups. To control for the injection route, the Control, DSS, and NaB groups received equal-volume intraperitoneal injections of sterile saline during the same intervention period. All interventions were maintained for 7 days. Thereafter, all groups were re-exposed to 2.5% DSS for another 7 days, while the corresponding NaB, PDTC, or combined PDTC+NaB interventions were continued in the assigned treatment groups. Thus, NaB treatment lasted 14 days in total, spanning the 7-day post-induction treatment phase and the subsequent 7-day DSS re-challenge. This schedule is consistent with a previously reported 14-day oral NaB regimen in mice (18). On day 22, mice were deeply anesthetized with isoflurane inhalation until loss of the pedal withdrawal reflex. After confirmation of deep anesthesia, mice were euthanized by cervical dislocation before blood, mesenteric lymph node, and colonic tissue collection. No injectable anesthetic was used for this terminal procedure. The use of deep inhalational anesthesia before euthanasia was intended to ensure unconsciousness and minimize pain or distress during terminal sampling. For Experiment 2, three mice per group were randomly selected using the same procedure. Samples from the same three mice were used for microbiome, Western blotting, immunofluorescence, and flow cytometry analyses.

### 2.6 Disease activity index assessment

Body weight was recorded daily at fixed time points from the initial DSS administration to the experimental endpoint, while fecal consistency and rectal bleeding were monitored and documented. DAI served to quantitatively evaluate colitis severity and was computed as follows: DAI = weight loss score + stool consistency score + rectal bleeding score, with scoring criteria adapted from Wu et al. (19). Weight loss was scored by reduction extent: no loss, 0; 1%–5%, 1; 6%–10%, 2; 11%–20%, 3; and >20%, 4. Stool consistency was graded as follows: well-formed pellets, 0; loose and unformed stools, 2; and watery diarrhea, 4. Rectal bleeding was scored as follows: no blood, 0; occult blood positive, 2; gross bleeding, 3; and gross rectal bleeding with active hemorrhage, 4.

### 2.7 Histopathological analysis of colonic tissue

Approximately 2 cm of mid-colonic tissue was excised, underwent fixation in 4% paraformaldehyde solution for 24 h, dehydration using a graded ethanol series, clearing with xylene, and embedding in paraffin. The embedded specimens underwent cutting into serial 4-μm sections, followed by deparaffinization, rehydration, and hematoxylin and eosin staining. Sections were mounted with neutral resinous medium, and histomorphological examination and image acquisition were conducted using a Leica optical microscope. Two pathologists, blinded to group assignment, independently assessed the sections, concentrating on inflammatory cell infiltration and mucosal ulceration. Tissue injury was scored according to Schmidt et al. (20).

### 2.8 ELISA

Colonic tissue (100 mg) underwent homogenization in radioimmunoprecipitation assay lysis buffer comprising 1% phenylmethylsulfonyl fluoride and a protease inhibitor cocktail, then centrifuged at 12,000 rpm for 5 min at 4°C to obtain the supernatant. Protein concentrations were ascertained via the bicinchoninic acid method, with all samples standardized to 3,000 ng/mL. Blood samples were centrifuged to separate serum before analysis. Per the supplier’s protocols, cytokine levels in colonic homogenates and serum were measured in parallel, and pro-inflammatory cytokines (IL-17A, IL-21, and IL-22) and anti-inflammatory/immunoregulatory cytokines (IL-10 and TGF-β1) were quantified using standard curves.

### 2.9 Western blot detection

Total protein was extracted from colon tissues and quantified using a BCA assay kit (Biosharp, BL521A). For each biological replicate, 10 μg of protein was loaded per lane, separated by SDS-PAGE, and transferred onto PVDF membranes (Merck Millipore, IPVH00010). After blocking with QuickBlock™ Western blocking buffer (Beyotime, P0252), membranes were incubated overnight at 4°C with primary antibodies against p-IκBα (Proteintech, 82349-1-RR, 1:2000), IκBα (Proteintech, 10268-1-AP, 1:10000), p-p65 (Proteintech, 82335-1-RR, 1:5000), p65 (Proteintech, 10745-1-AP, 1:4000), Claudin-1 (Proteintech, 28674-1-AP, 1:5000), Occludin (Proteintech, 27260-1-AP, 1:10000), ZO-1 (Proteintech, 21773-1-AP, 1:5000), and GAPDH (Proteintech, 60004-1-Ig, 1:50000). Membranes were then incubated with HRP-conjugated goat anti-rabbit or goat anti-mouse secondary antibodies (Proteintech, 1:10000) at 37°C for 1 h. Signals were detected using Meilunbio^®^ ECL substrate (MA0186) for 5 min in the dark and visualized with a chemiluminescence imaging system (SCG-W3000). Band intensities were quantified using ImageJ. Protein levels were normalized to GAPDH, and p-IκBα and p-p65 were additionally normalized to total IκBα and p65, respectively. Relative values were normalized to the blank/control group. Three biological replicates were included per group.

### 2.10 Immunofluorescence

Paraffin sections measuring 4 μm in thickness were generated following the methodology outlined in Section 2.7. Following deparaffinization and sequential hydration, antigen retrieval was executed through heating sections in citrate buffer (pH 6.0) for 10 min. After bovine serum albumin blocking, sections underwent incubation with primary antibodies targeting Claudin-1, Occludin, and ZO-1 overnight at 4°C. Subsequently, sections were exposed to respective fluorescent secondary antibodies for 30 min under dark conditions. Thereafter, nuclei underwent counterstaining with 4’,6-diamidino-2-phenylindole (DAPI), and sections received mounting utilizing anti-fade mounting medium. Images were captured under a fluorescence microscope (Leica DM2500, Wetzlar, Germany).

### 2.11 Flow cytometry

Mesenteric lymph nodes and spleens were minced, digested with 0.2% collagenase II (Servicebio, C8150) at 37°C for 10–20 and 20–30 min, respectively, and filtered to obtain single-cell suspensions. Splenic erythrocytes were lysed with red blood cell lysis buffer (Meilunbio, MA0207) for 5 min. A total of 1 × 10^6^ cells per sample were stimulated with PMA (50 ng/mL) and ionomycin (500 ng/mL) for 2 h, followed by brefeldin A (2 μg/mL) for another 2 h at 37°C with 5% CO_2_. Cells were blocked with anti-mouse CD16/CD32 [clone 2.4G2] (Cell Signaling Technology, 88280) for 20 min and surface-stained with PE anti-mouse CD4 [clone GK1.5] (Elabscience, E-AB-F1097D) and PE/Cyanine7 anti-mouse CD25 [clone PC-61.5.3] (Elabscience, E-AB-F1102H) on ice for 20 min. After fixation and permeabilization (Elabscience, E-CK-A109), cells were stained intracellularly with APC anti-mouse IL-17A [clone TC11-18H10.1] (Elabscience, E-AB-F1199E) and FITC anti-mouse/rat Foxp3 [clone FJK-16s] (Elabscience, E-AB-F1351C) for 30 min. Single-stained controls were used for fluorescence compensation. No viability dye was included; therefore, nonviable cells could not be reliably excluded. Sequential gating selected stable events, lymphocytes, singlets, and CD4^+^ cells. Th17 and Treg cells were defined as CD4^+^IL-17A^+^ and CD4^+^CD25^+^Foxp3^+^ cells, respectively. Full-resolution gating plots are provided in **Supplementary Figure 1**. Data were acquired using a Raise Cyte flow cytometer and analyzed with FlowJo v7.6.1.

### 2.12 Genomic DNA extraction and 16S-rRNA sequencing of feces

Colonic contents were collected under sterile conditions and sent to Novogene Bioinformatics Technology Co., Ltd. (Tianjin, China) for 16S rRNA sequencing. Total genomic DNA was extracted, and the V3-V4 region of the bacterial 16S rRNA gene was amplified using barcode-fused primers. Amplicon libraries were sequenced on the NovaSeq 6000 platform in PE250 mode. Raw paired-end reads were quality-filtered using Trimmomatic v0.36 with a 50-bp sliding window, a Q20 average-quality threshold, and a minimum retained length of 120 bp. Reads containing ambiguous bases were removed using PEAR v0.9.6. Paired-end reads were merged using FLASH v1.20 and PEAR, with a minimum overlap of 10 bp and a maximum mismatch rate of 0.1. Chimeric and short non-qualified sequences were removed using Vsearch v2.7.1/UCHIME to obtain clean tags. Across samples, 59,401-98,589 clean tags were retained per sample. Clean tags were clustered into operational taxonomic units (OTUs) at 97% sequence similarity using UPARSE/uclust. Representative OTU sequences were taxonomically annotated using the RDP Classifier against the SILVA 128 database, with a confidence threshold of 0.7. OTU tables were rarefied to 59,401 tags per sample before downstream alpha- and beta-diversity analyses, which were performed using QIIME/QIIME2 scripts and R.

### 2.13 Targeted SCFAs detection

The reaction mixture was diluted with 50% acetonitrile to reach a final volume of 1,000 μL and analyzed by liquid chromatography-electrospray ionization tandem mass spectrometry. SCFA quantification was conducted using an ExionLC AD ultra-high performance liquid chromatography system connected to an AB SCIEX QTRAP 6500+ mass spectrometer, employing acetic acid, propionic acid, butyric acid, isovaleric acid, valeric acid, and hexanoic acid as reference standards. Chromatographic separation was accomplished utilizing a Waters BEH C18 column (150 × 2.1 mm, 1.7 μm) held at 40°C with an injection volume of 2 μL. Mobile phase A contained 0.1% formic acid in water, whereas mobile phase B consisted of acetonitrile containing 0.1% formic acid. Mass spectrometry parameters were as follows: curtain gas pressure, 35 psi; collision gas, medium level; spray voltage, −4,500 V; and ion source temperature, 450°C. Standard curve equations were generated by linear fitting of peak areas for each SCFA standard against the corresponding concentrations. SCFA concentrations in samples were determined by inserting test-sample peak areas into the regression equations.

### 2.14 Statistical analysis

Gut microbiota analyses were performed using the Majorbio Cloud Platform. Other data were analyzed using GraphPad Prism v10.0 and are presented as mean ± SEM. Body-weight and DAI data were analyzed using two-way repeated-measures ANOVA with Bonferroni correction. Multiple-group comparisons were performed using one-way ANOVA followed by Tukey’s or Bonferroni’s test; Welch’s ANOVA with Dunnett’s T3 test was used for unequal variances. Alpha diversity was analyzed using the Kruskal–Wallis test, and beta diversity using PERMANOVA/Adonis. P values from pairwise ANOSIM and SCFA analyses were adjusted using the Benjamini–Hochberg false-discovery-rate procedure and reported as q values. All tests were two-tailed, and P < 0.05 or q < 0.05 was considered statistically significant.

## 3 Results

### 3.1 FMT attenuated clinical symptoms and colonic tissue injury in DSS-induced UC mice

**Figure 1:**
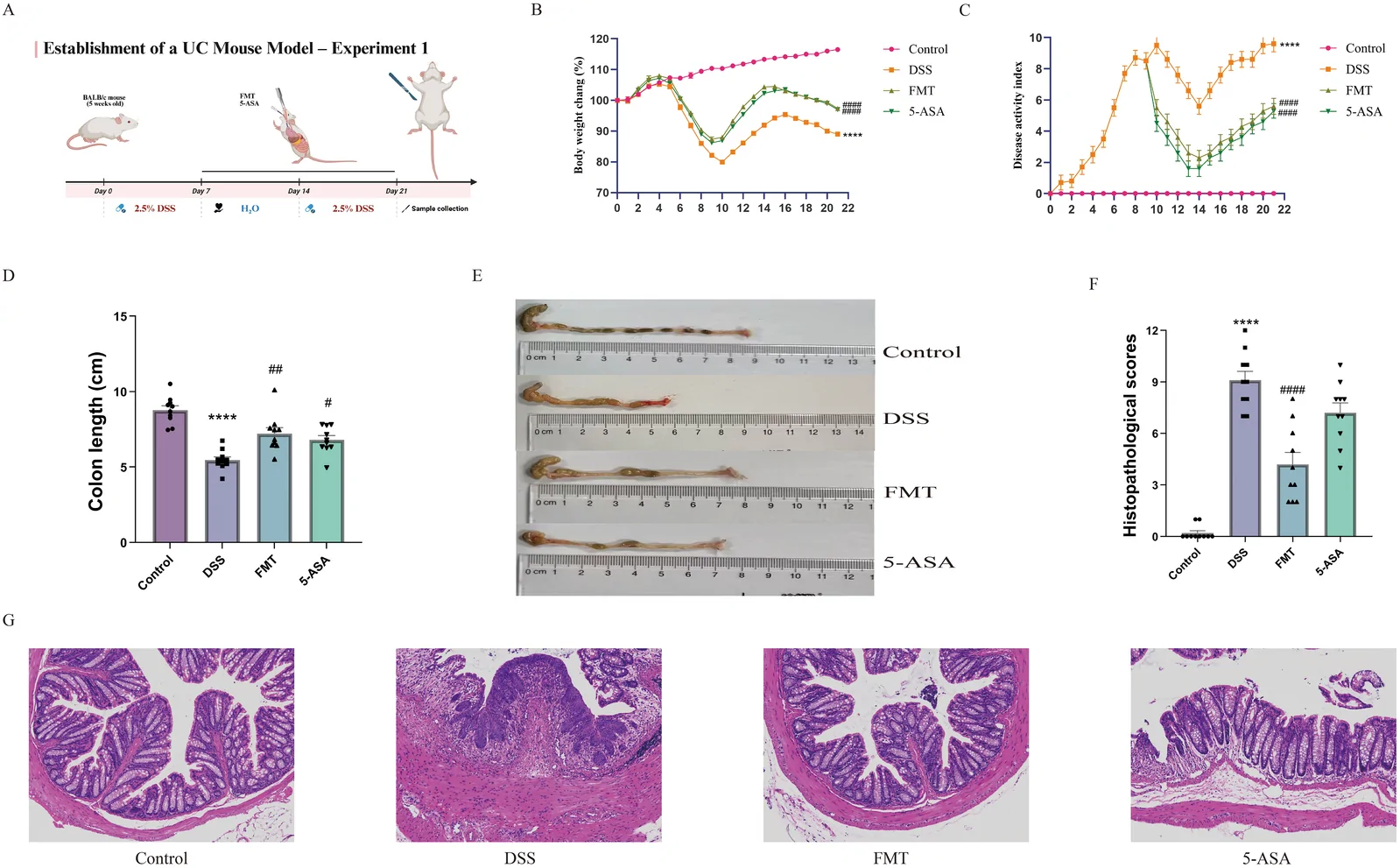
FMT alleviates clinical symptoms and colonic tissue injury in DSS-induced UC mice. **(A)** Experimental design. **(B)** Body weight changes. **(C)** Disease activity index (DAI) scores. **(D, E)** Colon length quantification and representative images. **(F)** Histopathological injury scores. **(G)** Representative hematoxylin and eosin (H&E)-stained colon sections. Scale bars (200 μm) are shown in the images; original magnification, ×100. Fecal microbiota transplantation (FMT) alleviated DSS-induced weight loss, reduced DAI scores, prevented colon shortening, and improved mucosal injury. 5-aminosalicylic acid (5-ASA) was used as a positive control. For **(B, C, D, F)**, n = 10 biologically independent mice per group. Data are presented as mean ± standard error of the mean (SEM). Body weight and DAI were analyzed using two-way repeated-measures ANOVA with Bonferroni correction; colon length and histopathological scores were analyzed using one-way ANOVA with Bonferroni correction. ****P < 0.0001 versus the Control group; ^#^*P* < 0.05, ^##^*P* < 0.01, and ^####^*P* < 0.0001 versus the DSS group. **(E, G)** Show representative images. UC, ulcerative colitis.

The overall workflow for model establishment, treatment, and sample processing is shown in **Figure 1A**. Compared with the healthy control group, DSS-induced UC mice exhibited marked body weight loss (**Figure 1B**), increased DAI scores (**Figure 1C**), shortened colon length (**Figures 1D, E**), and elevated histopathological injury scores (**Figure 1F**). Histopathological analysis further showed mucosal epithelial erosion, ulcer formation, and inflammatory cell infiltration in the colonic tissues of UC mice (**Figure 1G**). Compared with the DSS group, mice treated with FMT or 5-ASA showed significantly improved body weight (**Figure 1B**), reduced DAI scores (**Figure 1C**), and increased colon length (**Figures 1D, E**). In addition, FMT and 5-ASA preserved mucosal integrity, with only mild inflammatory cell infiltration observed (**Figure 1G**), thereby significantly reducing histopathological injury scores (**Figure 1F**).

### 3.2 FMT improved the inflammatory status of DSS-induced UC mice

**Figure 2:**
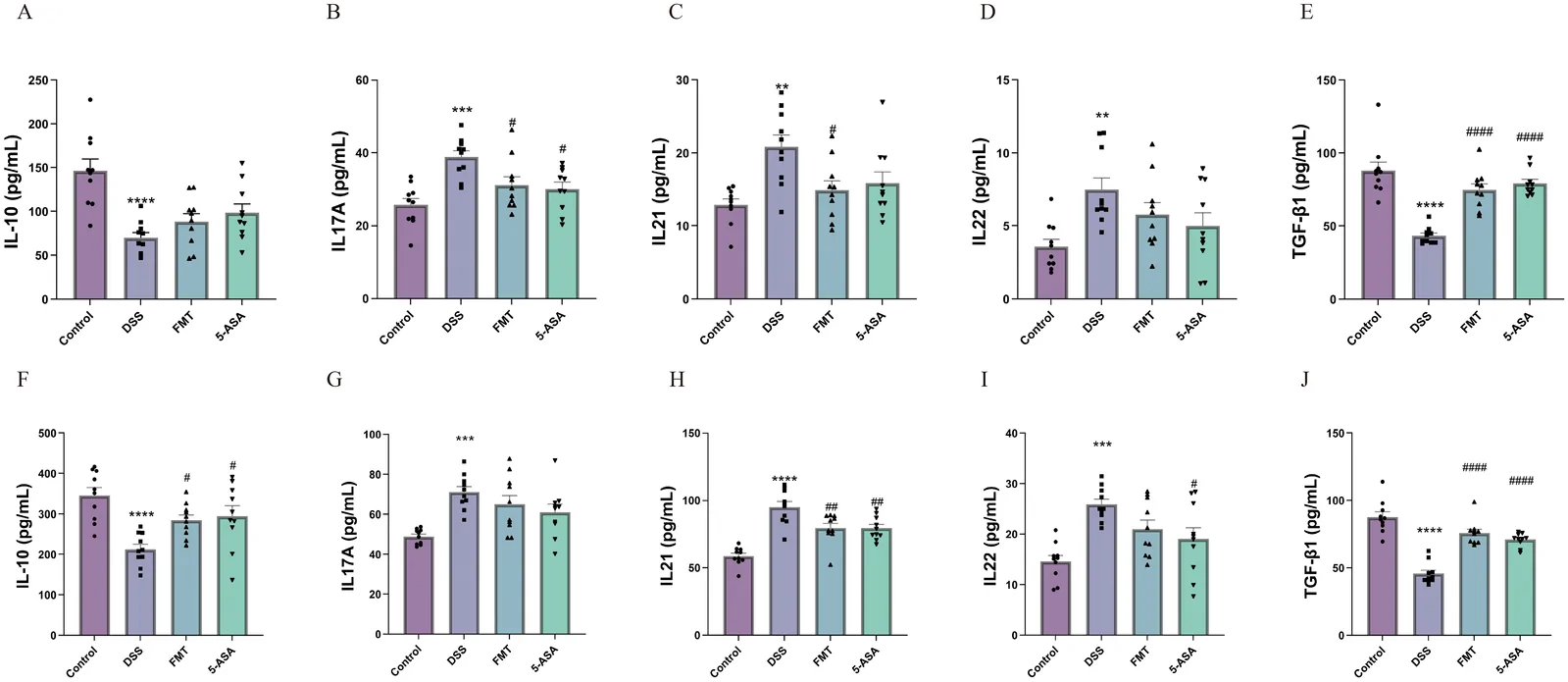
FMT improves the inflammatory status in DSS-induced UC mice. **(A–E)** Levels of IL-10, IL-17A, IL-21, IL-22, and TGF-β1 in colonic tissues. **(F–J)** Serum levels of these cytokines. FMT was associated with changes in selected pro- and anti-inflammatory cytokine concentrations in colonic tissues and serum. 5-aminosalicylic acid (5-ASA) showed similar effects. For all cytokine measurements, n = 10 biologically independent mice per group. Data are presented as mean ± standard error of the mean (SEM). Group differences were analyzed using ordinary one-way analysis of variance (ANOVA) followed by Tukey’s multiple-comparisons test. All reported P values are Tukey-adjusted P values. *P < 0.05, **P < 0.01, ***P < 0.001, and ****P < 0.0001 versus the Control group; ^#^*P* < 0.05, ^##^*P* < 0.01, and ^####^*P* < 0.0001 versus the DSS group. UC, ulcerative colitis.

As shown in **Figures 2A–E**, compared with the control group, the DSS group showed significantly increased levels of the pro-inflammatory cytokines IL-17A (*P* < 0.001), IL-21 (*P* < 0.01), and IL-22 (*P* < 0.01) in colonic tissues, whereas the anti-inflammatory cytokines IL-10 (*P* < 0.0001) and TGF-β1 (*P* < 0.0001) were markedly decreased. After intervention, colonic IL-17A and IL-21 levels were significantly reduced in the FMT group (*P* < 0.05), while TGF-β1 levels were significantly increased (*P* < 0.0001). Similarly, the 5-ASA group showed decreased IL-17A levels (*P* < 0.05) and increased TGF-β1 levels (*P* < 0.0001).

As shown in **Figures 2F–J**, serum cytokine levels showed trends consistent with those observed in colonic tissues. The DSS group exhibited significantly increased serum levels of IL-17A (*P* < 0.001), IL-21 (*P* < 0.0001), and IL-22 (*P* < 0.001), accompanied by decreased levels of IL-10 (*P* < 0.0001) and TGF-β1 (*P* < 0.0001). After treatment, serum IL-21 was significantly reduced in the FMT group (*P* < 0.01), whereas IL-10 (*P* < 0.05) and TGF-β1 (*P* < 0.0001) were significantly increased. In the 5-ASA group, serum IL-21 (*P* < 0.01) and IL-22 (*P* < 0.05) levels were decreased, while IL-10 (*P* < 0.05) and TGF-β1 (*P* < 0.0001) levels were upregulated. These results indicate that both FMT and 5-ASA effectively suppressed DSS-induced overproduction of pro-inflammatory cytokines while enhancing anti-inflammatory cytokine levels, suggesting that both interventions modulated the inflammatory microenvironment and exerted potential immunoprotective effects.

### 3.3 FMT restored colonic mucosal integrity in DSS-induced UC mice

**Figure 3:**
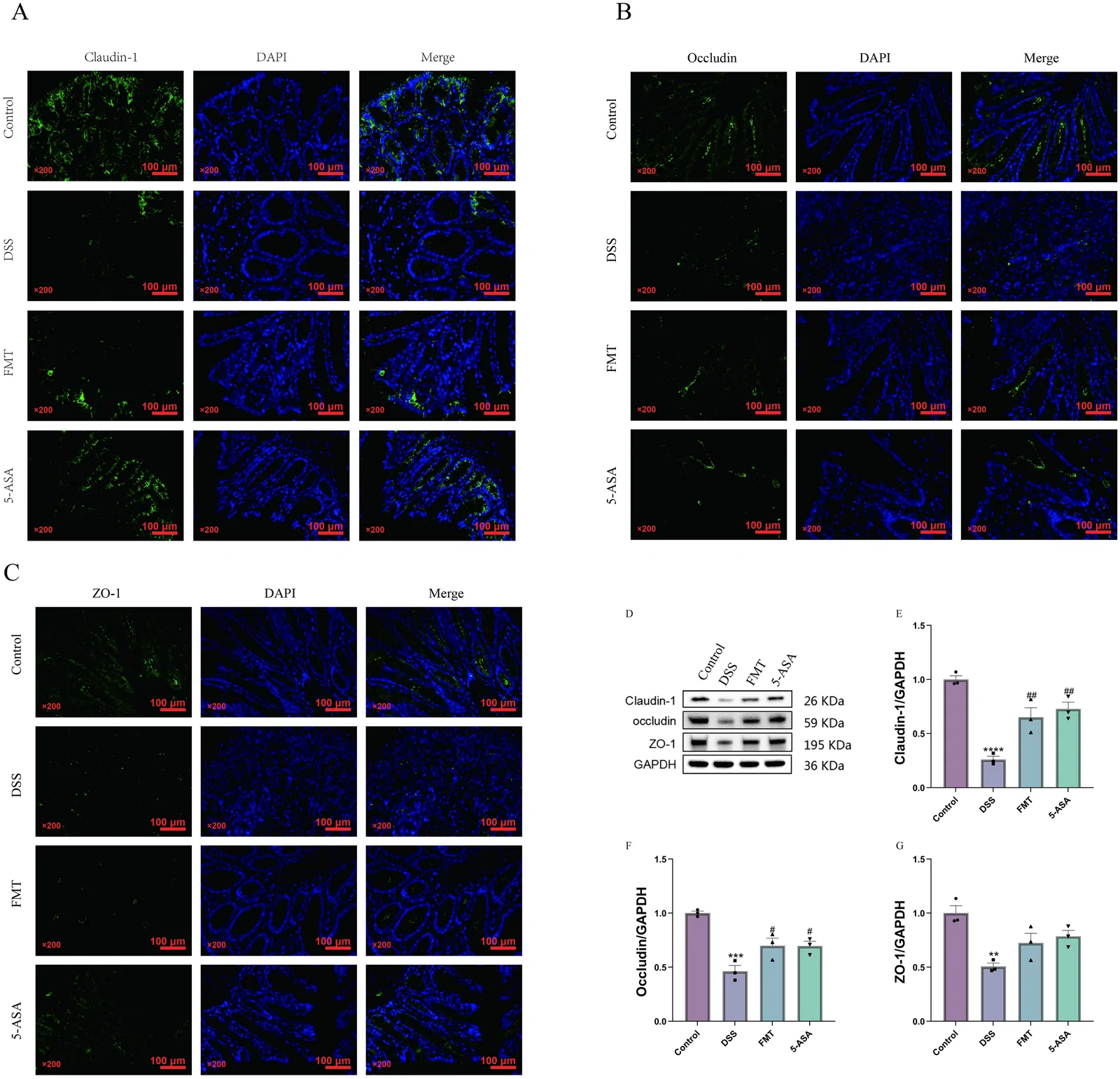
FMT restores colonic mucosal barrier integrity in DSS-induced UC mice. **(A–C)** Representative immunofluorescence images of Claudin-1, Occludin, and zonula occludens-1 (ZO-1) in colonic tissues from the Control, DSS, FMT, and 5-ASA groups. Green fluorescence indicates positive staining, and blue fluorescence indicates 4′,6-diamidino-2-phenylindole (DAPI)-stained nuclei. Scale bars (100 μm) are shown; original magnification, ×200. **(D)** Representative Western blot bands of Claudin-1, Occludin, and ZO-1, with GAPDH as the loading control. **(E–G)** Densitometric quantification of Claudin-1/GAPDH, Occludin/GAPDH, and ZO-1/GAPDH, respectively. 5-aminosalicylic acid (5-ASA) was used as a positive control. **(A–D)** show representative images or immunoblots from n = 3 biologically independent samples per group. For **(E–G)**, data are presented as the mean ± standard error of the mean (SEM), with n = 3 biologically independent samples per group. Group differences were analyzed using ordinary one-way analysis of variance (ANOVA) followed by Tukey’s multiple-comparisons test. All reported P values are Tukey-adjusted P values. **P < 0.01, ***P < 0.001, and ****P < 0.0001 versus the Control group; ^#^P < 0.05 and ^##^P < 0.01 versus the DSS group. UC, ulcerative colitis.

Next, IHC/IF and WB were performed to evaluate the expression of the tight junction-related proteins Claudin-1, Occludin, and ZO-1 in intestinal epithelial tissues from each treatment group. After DAPI counterstaining, nuclei exhibited blue fluorescence under ultraviolet excitation, whereas positive signals for Claudin-1, Occludin, and ZO-1 appeared as green fluorescence (**Figures 3A–C**). The control group showed continuous and intense green fluorescence along the intestinal epithelium. In contrast, the DSS group exhibited markedly weakened green fluorescence (**Figures 3A–C**). Consistently, WB analysis showed that the protein expression levels of Claudin-1 (*P* < 0.0001), Occludin (*P* < 0.001), and ZO-1 (*P* < 0.01) were significantly decreased, indicating DSS-induced disruption of tight junction proteins (**Figures 3D–G**).

After FMT or 5-ASA intervention, the fluorescence signals of Claudin-1, Occludin, and ZO-1 in the intestinal epithelium were markedly enhanced (**Figures 3A–C**). WB analysis further confirmed that Claudin-1 (*P* < 0.01) and Occludin (*P* < 0.05) protein expression levels were significantly upregulated (**Figures 3D–G**). These findings suggest that FMT and 5-ASA significantly ameliorated DSS-induced intestinal epithelial tight junction injury and helped maintain mucosal barrier integrity.

### 3.4 FMT reduces the colonic p-p65/p65 ratio in DSS-induced UC mice

**Figure 4:**
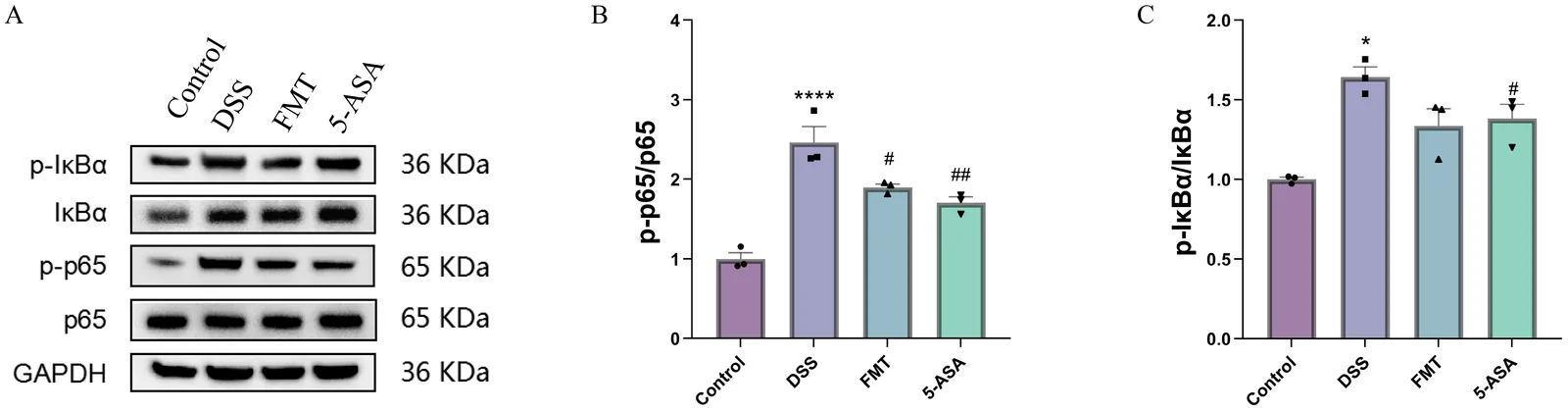
FMT reduces the colonic p-p65/p65 ratio in DSS-induced UC mice. **(A)** Representative Western blot bands of p-IκBα, IκBα, p-p65, and p65 in colonic tissues. **(B, C)** Quantification of the p-p65/p65 and p-IκBα/IκBα ratios. For **(B, C)**, n = 3 biologically independent samples per group. Data are presented as mean ± standard error of the mean (SEM). Group differences were analyzed using one-way ANOVA with Bonferroni correction or Dunnett’s T3 multiple-comparisons test when variances were unequal. *P < 0.05 and ****P < 0.0001 versus the Control group; ^#^*P* < 0.05 and ^##^*P* < 0.01 versus the DSS group. UC, ulcerative colitis.

As illustrated in **Figures 4A–C**, DSS treatment significantly increased the colonic p-IκBα/IκBα and p-p65/p65 ratios relative to the control group. Compared with the DSS group, FMT significantly reduced the p-p65/p65 ratio (*P* < 0.05), whereas 5-ASA significantly reduced both the p-IκBα/IκBα ratio (*P* < 0.05) and the p-p65/p65 ratio (*P* < 0.01).

### 3.5 FMT modulates Th17/Treg proportions in the spleen and mesenteric lymph nodes of DSS-induced UC mice

**Figure 5:**
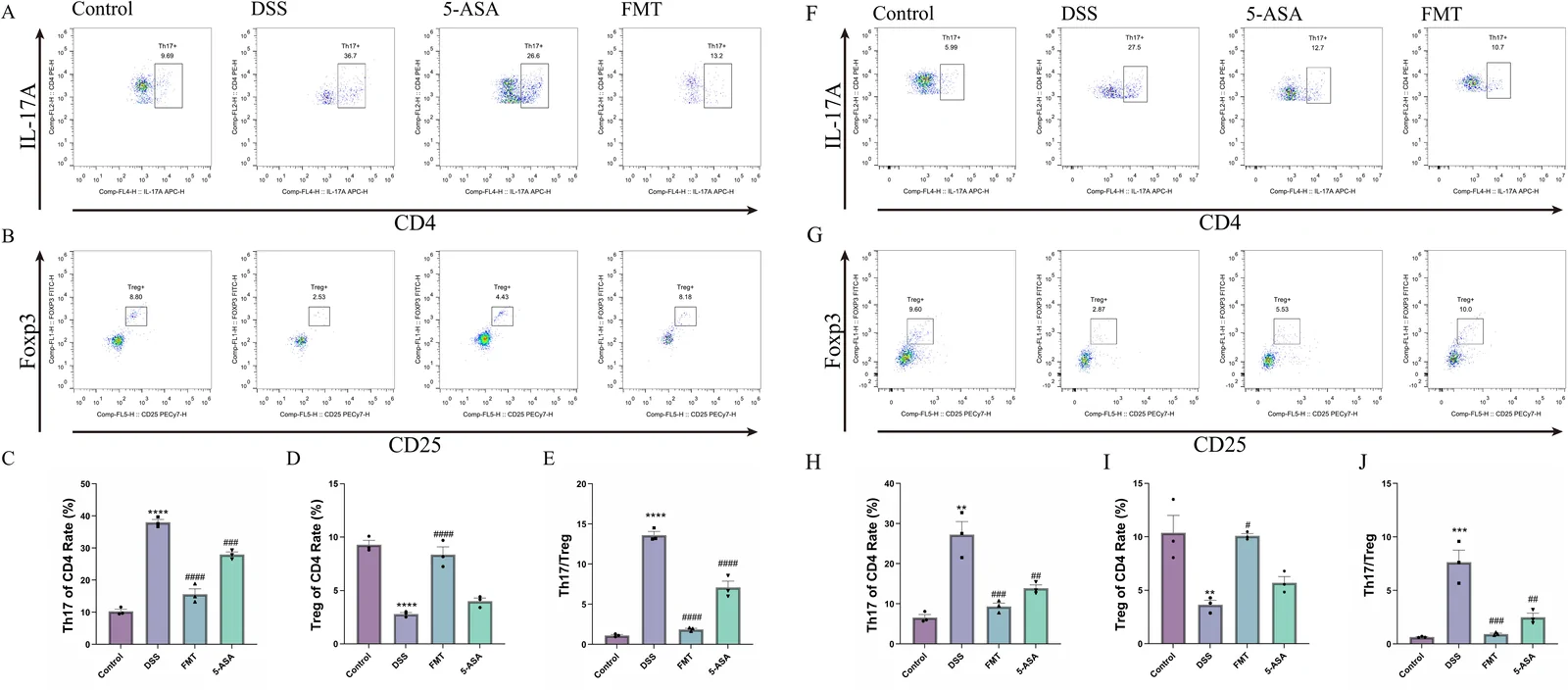
FMT modulates Th17/Treg proportions in the spleen and mesenteric lymph nodes of DSS-induced UC mice. **(A, B)** Representative flow cytometry plots of Th17 and Treg cells in spleen tissues. **(C–E)** Quantification of Th17 cells, Treg cells, and the Th17/Treg ratio. **(F, G)** Representative flow cytometry plots of Th17 and Treg cells in lymph nodes. **(H–J)** Quantification of Th17 cells, Treg cells, and the Th17/Treg ratio. Relative to the DSS group, FMT reduced Th17 proportions, increased Treg proportions, and reduced the Th17/Treg ratio in the spleen and mesenteric lymph nodes. 5-aminosalicylic acid (5-ASA) was used as a positive control. For **(C–E, H–J)**, n = 3 biologically independent samples per group. Data are presented as mean ± standard error of the mean (SEM). Group differences were analyzed using one-way ANOVA with Bonferroni correction or Dunnett’s T3 multiple-comparisons test when variances were unequal. **P < 0.01, and ****P < 0.0001 versus the Control group; ^#^*P* < 0.05, ^##^*P* < 0.01, ^###^*P* < 0.001, and ^####^*P* < 0.0001 versus the DSS group. UC, ulcerative colitis.

In splenic tissues, DSS markedly elevated Th17 cell proportions while reducing Treg cell proportions relative to the control group, with notable disruption of the Th17/Treg balance (all *P* < 0.0001). Relative to the DSS group, FMT treatment markedly reduced Th17 cell proportions, elevated Treg cell proportions, and reduced the Th17/Treg ratio in splenic tissues (all *P* < 0.0001). 5-ASA intervention also significantly decreased Th17 cell proportions (*P* < 0.001) and reduced the Th17/Treg ratio (*P* < 0.0001, **Figures 5A–E**; **Supplementary Figure 1** shows the full flow-cytometry gating strategy).

In lymph node tissues, DSS markedly elevated Th17 cell proportions, diminished Treg cell proportions, and increased the Th17/Treg ratio versus the control group (*P* < 0.01, *P* < 0.01, and *P* < 0.001, respectively). Relative to the DSS group, FMT intervention substantially reduced Th17 cell proportions, enhanced Treg cell proportions, and reduced the Th17/Treg ratio (*P* < 0.001, *P* < 0.05, and *P* < 0.001, respectively). 5-ASA intervention also significantly decreased Th17 cell proportions (*P* < 0.01) and reduced the Th17/Treg ratio (*P* < 0.001, **Figures 5F–J**).

Collectively, FMT and 5-ASA were associated with tissue-specific changes in Th17 and Treg proportions and the Th17/Treg ratio in the spleen and mesenteric lymph nodes relative to DSS.

### 3.6 FMT is associated with exploratory trends in colonic microbiota richness and community structure in DSS-induced UC mice

**Figure 6:**
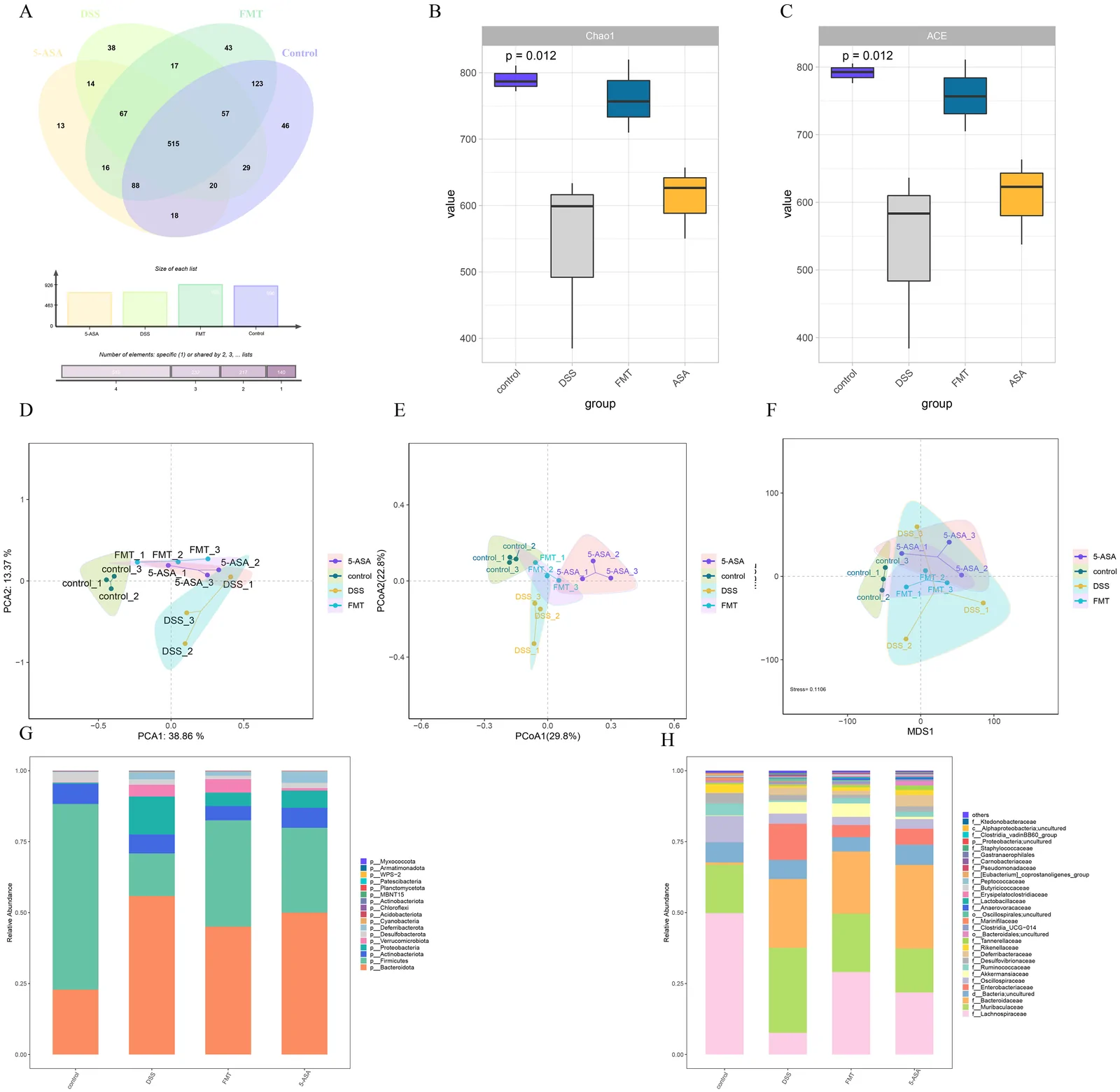
FMT-associated exploratory trends in colonic microbiota richness and community structure in DSS-induced UC mice. **(A)** Venn diagram showing OTU distribution and overlap among the Control, DSS, FMT, and 5-ASA groups. **(B, C)** Alpha-diversity indices (Chao1 and ACE) with Kruskal–Wallis test *P* values. **(D–F)** Beta-diversity analyses based on PCA, PCoA, and NMDS. **(G, H)** Relative abundances of colonic microbiota at the phylum and family levels. For **(A–H)**, n = 3 independent colonic-content samples per group. Alpha-diversity comparisons were performed using the Kruskal–Wallis test, with global *P* values shown. Community differences in **(D–F)** were assessed by PERMANOVA/Adonis. **(G, H)** Show descriptive microbial composition without statistical annotations. UC, ulcerative colitis.

Venn analysis revealed 896, 757, 926, and 751 detected OTUs in the Control, DSS, FMT, and 5-ASA groups, respectively. Among these OTUs, 515 were common to all four groups, whereas 46, 38, 43, and 13 OTUs were uniquely detected in the Control, DSS, FMT, and 5-ASA groups, respectively. Microbiome analysis included n = 3 samples per group. The FMT group showed greater OTUs overlap with the control group, suggesting a trend toward a control-like microbial profile (**Figure 6A**). α-diversity analysis showed that the Chao1 and ACE indices were reduced in the DSS group but increased after FMT and 5-ASA intervention (all P < 0.005. The indices were numerically higher in the FMT and 5-ASA groups than in the DSS group, suggesting an exploratory trend toward greater microbial richness (**Figures 6B, C**).

β-diversity analysis suggested treatment-associated differences in microbiota structure. At the OTU level, adonis/PERMANOVA showed an overall group effect (R^2^ = 0.4865, F = 2.5260, P = 0.007). However, genus-level adonis analysis was not significant (R^2^ = 0.4547, F = 2.2233, P = 0.065), and pairwise ANOSIM comparisons were not significant after BH correction. Thus, PCA, PCoA, and NMDS results were interpreted as community-level trends rather than definitive pairwise separation (**Figures 6D–F**).

Relative-abundance profiles suggested DSS-associated differences in dominant taxa, including Firmicutes, Bacteroidetes, Proteobacteria, Lachnospiraceae, Muribaculaceae, Bacteroidaceae, and Enterobacteriaceae (**Figures 6G, H**). Following FMT and 5-ASA intervention, several taxa showed apparent shifts toward the control profile. However, Wilcoxon testing did not identify significant DSS-versus-FMT differences at the tested phylum or genus levels after multiple-testing correction. Therefore, these taxon-level patterns are considered exploratory. Collectively, FMT- and 5-ASA-associated microbiota patterns should be interpreted as trends rather than evidence of partial restoration.

### 3.7 FMT increases endpoint fecal acetate and butyrate concentrations in DSS-induced UC mice

**Figure 7:**
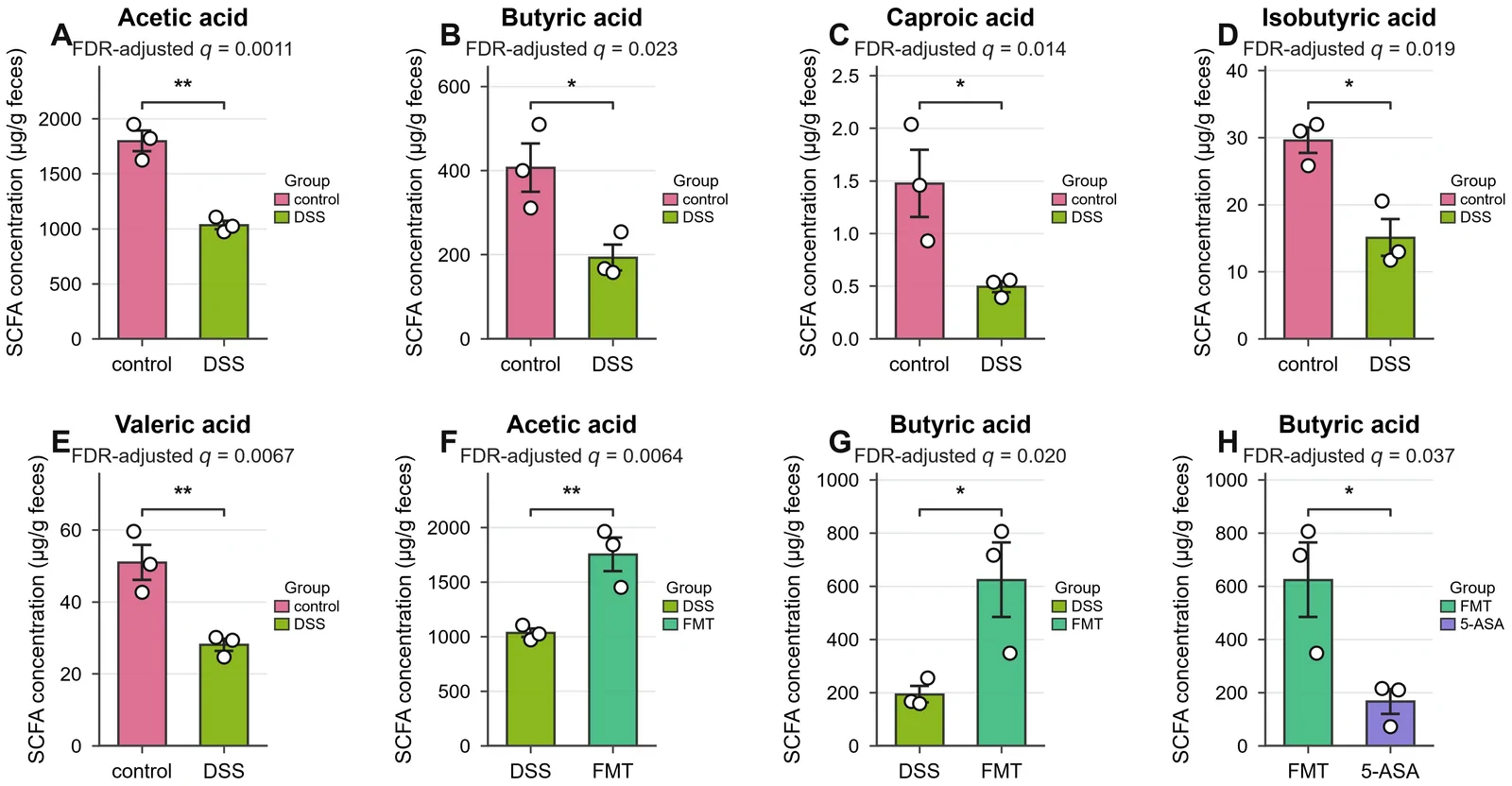
FMT increases endpoint fecal acetate and butyrate concentrations in DSS-induced UC mice. **(A–E)** Targeted quantification of fecal short-chain fatty acids (SCFAs) in the Control and dextran sulfate sodium (DSS) groups, including acetic acid (q = 0.0011), butyric acid (q = 0.023), caproic acid (q = 0.014), isobutyric acid (q = 0.019), and valeric acid (q = 0.0067). **(F, G)** Compared with the DSS group, fecal microbiota transplantation (FMT) significantly increased fecal acetic acid (q = 0.0064) and butyric acid (q = 0.020) concentrations. **(H)** Fecal butyrate concentrations were significantly higher in the FMT group than in the 5-aminosalicylic acid (5-ASA) group (q = 0.037). For each panel, n = 3 biologically independent fecal samples per group. Data are presented as the mean ± standard error of the mean (SEM). Pairwise comparisons were performed as described in the targeted metabolomics workflow. Raw P values were adjusted for multiple testing using the Benjamini–Hochberg false discovery rate (FDR) procedure, and the resulting adjusted P values (q values) are reported. q < 0.05 was considered statistically significant. The y-axis represents the fecal SCFA concentration (μg/g feces). *q < 0.05; **q < 0.01. UC, ulcerative colitis.

Targeted metabolite profiling showed that DSS-treated mice had lower endpoint fecal concentrations of multiple SCFAs than controls, including acetate (*P* = 0.0011), butyrate (*P* = 0.023), caproate (*P* = 0.014), isobutyrate (*P* = 0.019), and valerate (*P* = 0.0067; **Figures 7A–E**). Compared with DSS, FMT significantly increased endpoint fecal acetate (*P* = 0.0064; **Figure 7F**) and butyrate concentrations (*P* = 0.020; **Figure 7G**). Endpoint fecal butyrate concentration was also significantly higher in the FMT group than in the 5-ASA group (*P* = 0.037; **Figure 7H**). Given the small sample size, single endpoint, and lack of independent validation, these findings remain exploratory and do not establish butyrate production, metabolic flux, absorption, or restoration to baseline. Nevertheless, the significant group differences support further evaluation of endpoint fecal butyrate concentration as an FMT-associated readout in longitudinal and functional studies.

### 3.8 NaB reduces clinical symptoms and colonic tissue damage in DSS-induced UC mice

**Figure 8:**
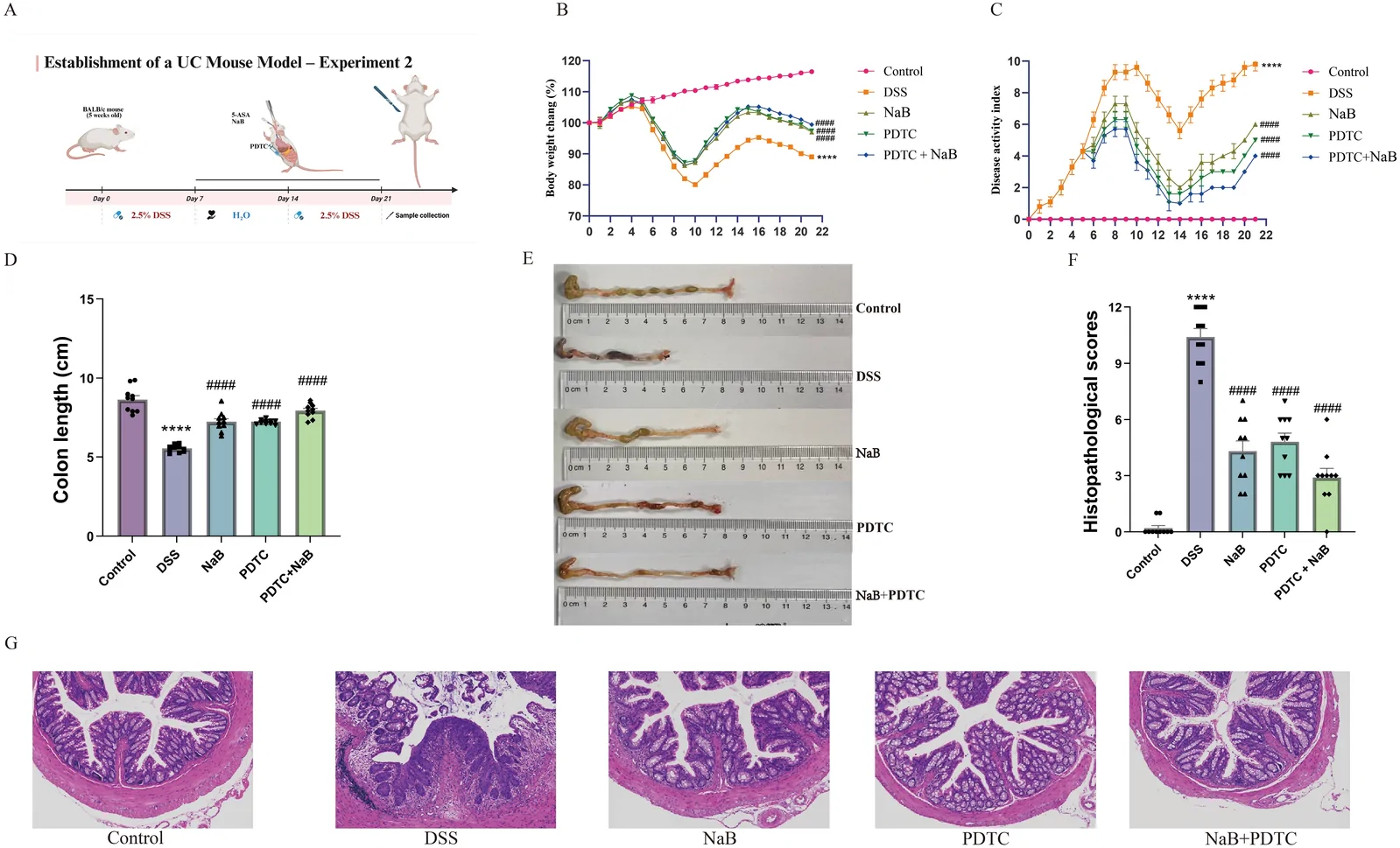
Butyrate alleviates clinical symptoms and colonic tissue injury in DSS-induced UC mice. **(A)** Experimental design. **(B)** Body weight changes. **(C)** Disease activity index (DAI) scores. **(D, E)** Colon length quantification and representative images. **(F)** Histopathological injury scores. **(G)** Representative hematoxylin and eosin (H&E)-stained colon sections. Scale bars (200 μm) are shown in the images; original magnification, ×100. Sodium butyrate (NaB), pyrrolidine dithiocarbamate (PDTC), and combined NaB + PDTC treatment alleviated DSS-induced weight loss, reduced DAI scores, prevented colon shortening, and improved colonic tissue injury. For **(B, C, D, F)**, n = 9 biologically independent mice per group. Data are presented as mean ± standard error of the mean (SEM). Body weight and DAI were analyzed using two-way repeated-measures ANOVA with Bonferroni correction; colon length and histopathological scores were analyzed using one-way ANOVA with Bonferroni correction. ****P < 0.0001 versus the Control group; ^####^*P* < 0.0001 versus the DSS group. **(E, G)** show representative images. UC, ulcerative colitis.

The modeling and drug intervention workflow is shown in **Figure 8A**. Relative to the healthy control group, DSS-induced mice demonstrated marked body weight loss (*P* < 0.0001, **Figure 8B**), exhibiting the most pronounced decrease on day 10; body weight continued to remain below control group levels through the experimental endpoint. After 14 days of NaB, PDTC, or combined intervention, the body weight change rate significantly recovered (*P* < 0.0001, **Figure 8B**). DAI in the DSS group demonstrated a significant elevation from day 1 (*P* < 0.01, **Figure 8C**), reached its peak on day 9, and maintained consistently elevated levels versus the control group (*P* < 0.0001). After drug intervention, DAI decreased markedly (*P* < 0.0001, **Figure 8C**). Colonic length exhibited a notable decrease in the DSS group, measuring 5.54 ± 0.79 cm versus 8.65 ± 0.58 cm in the control group (*P* < 0.0001, **Figure 8D**). After intervention, colonic length recovered to 7.24 ± 1.02 cm, 7.24 ± 0.8 cm, and 7.95 ± 0.75 cm in the NaB, PDTC, and combination groups, respectively (*P* < 0.0001, **Figure 8D**), and gross colon morphology is presented in **Figure 8E**. Histopathological analysis showed that the control group preserved intact colonic architecture with only mild abnormalities (**Figures 8F, G**), whereas the DSS group had extensive ulcerative necrosis, crypt destruction, and inflammatory cell infiltration. Histopathological injury scores were significantly lower in the NaB, PDTC, and combination groups than in the DSS group (all *P* < 0.0001; **Figure 8F**). Representative sections from each intervention group showed less extensive epithelial injury and inflammatory-cell infiltration than the DSS group (**Figure 8G**). These findings describe comparisons with the DSS group only.

### 3.9 NaB alleviates the inflammatory status in DSS-induced UC mice

**Figure 9:**
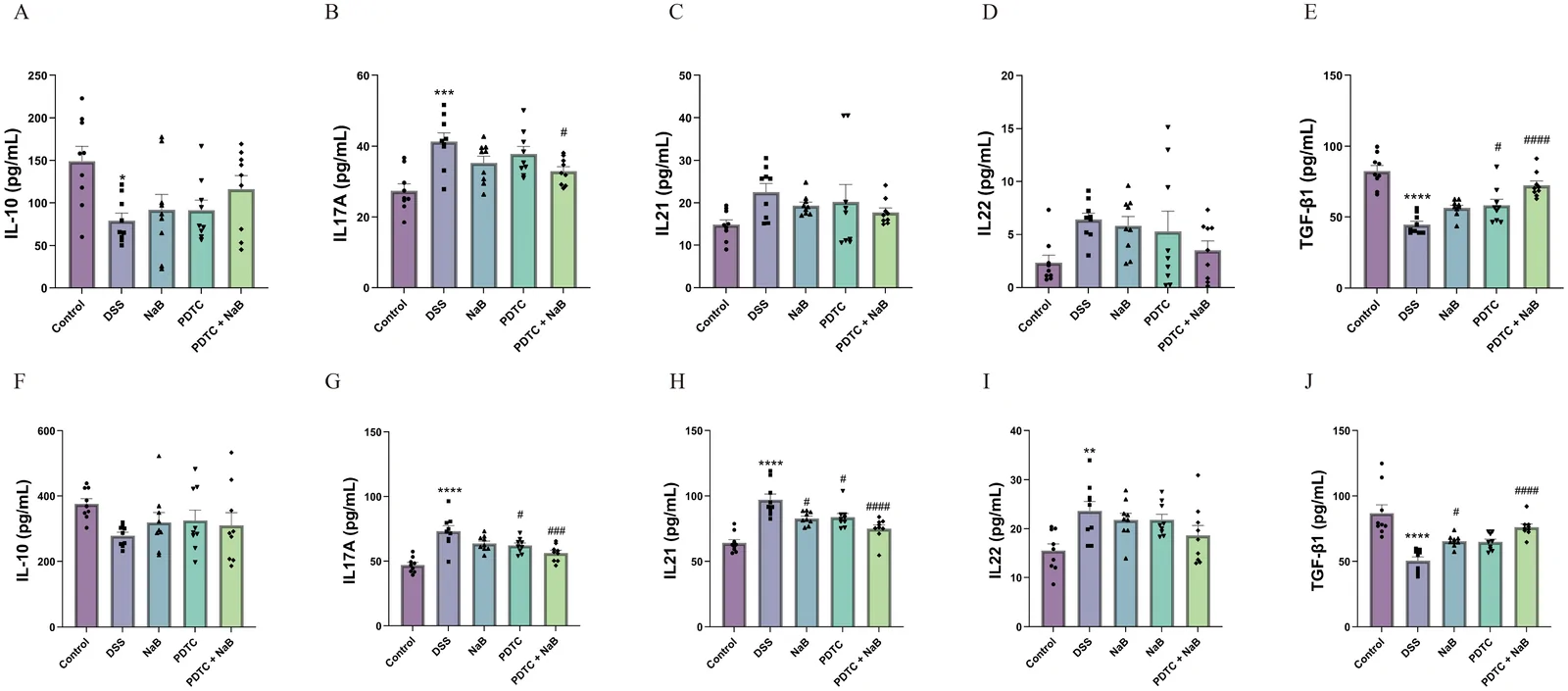
Cytokine concentrations after NaB, PDTC, and combined NaB + PDTC treatment in DSS-induced UC mice. **(A–E)** Levels of IL-10, IL-17A, IL-21, IL-22, and TGF-β1 in colonic tissues. **(F–J)** Serum levels of these cytokines. Relative to the DSS group, treatment with NaB, PDTC, or their combination was associated with changes in selected colonic and serum cytokine concentrations. The combination was not directly compared with either monotherapy. For all cytokine measurements, n = 9 biologically independent mice per group. Data are presented as mean ± standard error of the mean (SEM). Group differences were analyzed using ordinary one-way analysis of variance (ANOVA) followed by Tukey’s multiple-comparisons test. All reported *P* values are Tukey-adjusted *P* values. *P < 0.05, **P < 0.01, ***P < 0.001, and ****P < 0.0001 versus the Control group; ^#^*P* < 0.05, ^###^*P* < 0.001, and ^####^*P* < 0.0001 versus the DSS group.

Colonic concentrations of five cytokines, including IL-10, IL-17A, IL-21, IL-22 and TGF-β1, were quantified (**Figures 9A–E**). Compared with the Control group, DSS-treated mice showed a decreased colonic IL-10 concentration (*P* < 0.05; **Figure 9A**), whereas the colonic IL-17A concentration was increased (*P* < 0.001; **Figure 9B**) and the TGF-β1 concentration was decreased (*P* < 0.0001; **Figure 9E**). After 14 days of intervention, PDTC treatment increased colonic TGF-β1 relative to the DSS group (*P* < 0.05; **Figure 9E**). Combined PDTC and NaB treatment decreased colonic IL-17A (*P* < 0.05; **Figure 9B**) and increased TGF-β1 (*P* < 0.0001; **Figure 9E**). Serum concentrations of the same five cytokines were also measured (**Figures 9F–J**). Compared with the Control group, DSS-treated mice exhibited increased serum concentrations of IL-17A (*P* < 0.0001; **Figure 9G**), IL-21 (*P* < 0.0001; **Figure 9H**) and IL-22 (*P* < 0.01; **Figure 9I**), together with a decreased TGF-β1 concentration (*P* < 0.0001; **Figure 9J**). Relative to the DSS group, NaB treatment decreased serum IL-21 (*P* < 0.05; **Figure 9H**) and increased TGF-β1 (*P* < 0.05; **Figure 9J**). PDTC treatment decreased serum IL-17A and IL-21 concentrations (both *P* < 0.05; **Figures 9G,H**). Combined PDTC and NaB treatment decreased IL-17A (*P* < 0.001; **Figure 9G**) and IL-21 (*P* < 0.0001; **Figure 9H**), while increasing TGF-β1 (*P* < 0.0001; **Figure 9J**). Collectively, NaB, PDTC, and combined NaB/PDTC treatment were associated with changes in selected cytokine concentrations relative to the DSS group. Because the combination was not directly compared with either monotherapy, these results should be interpreted only as comparisons with the DSS group.

### 3.10 NaB restores colonic mucosal integrity in DSS-induced UC mice

**Figure 10:**
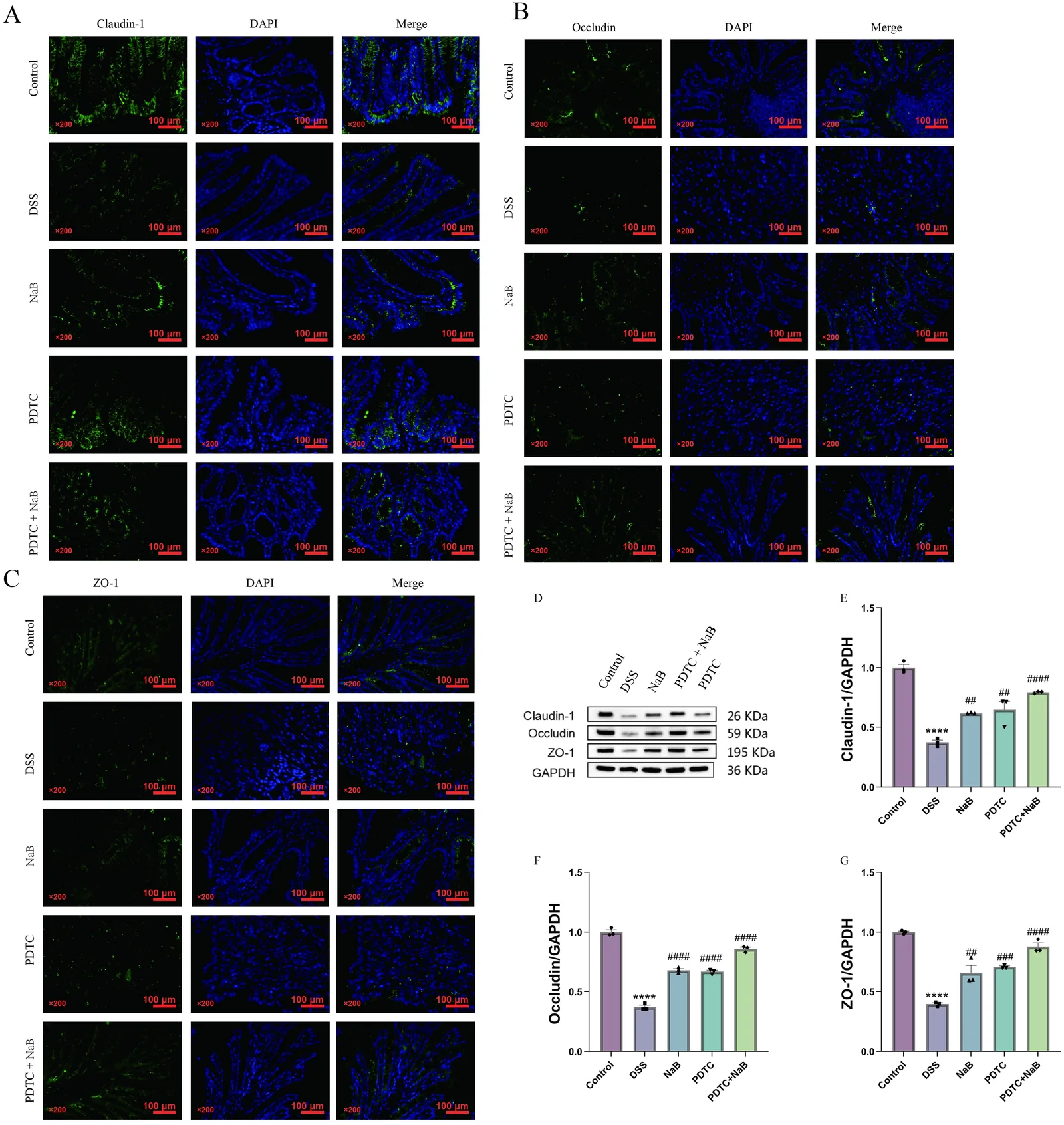
Butyrate restores colonic mucosal barrier integrity in DSS-induced UC mice. **(A–C)** Representative immunofluorescence images of Claudin-1, Occludin, and zonula occludens-1 (ZO-1) in colonic tissues from the Control, DSS, NaB, PDTC, and PDTC + NaB groups. Green fluorescence indicates positive staining, and blue fluorescence indicates 4′,6-diamidino-2-phenylindole (DAPI)-stained nuclei. Scale bars (100 μm) are shown in the images; original magnification, ×200. **(D)** Representative Western blot bands of Claudin-1, Occludin, and ZO-1, with GAPDH as the loading control. **(E–G)** Densitometric quantification of Claudin-1/GAPDH, Occludin/GAPDH, and ZO-1/GAPDH, respectively. **(A–D)** Show representative images or immunoblots from n = 3 biologically independent samples per group. For **(E–G)**, data are presented as the mean ± standard error of the mean (SEM), with n = 3 biologically independent samples per group. Group differences were analyzed using one-way analysis of variance (ANOVA), followed by Bonferroni’s multiple-comparisons test when variances were homogeneous or Dunnett’s T3 test when variances were unequal. All reported P values were adjusted for multiple comparisons using the applicable *post hoc* procedure. **P < 0.0001 versus the Control group; ^##^*P* < 0.01, ^###^*P* < 0.001, and ^####^*P* < 0.0001 versus the DSS group. UC, ulcerative colitis.

To assess intestinal epithelial tight junction protein expression, IF/immunohistochemistry was employed to detect Claudin-1 (**Figure 10A**), Occludin (**Figure 10B**), and ZO-1 (**Figure 10C**). DAPI counterstaining showed blue nuclear fluorescence, whereas Claudin-1, Occludin, and ZO-1 displayed green fluorescence. In the control group, strong continuous green fluorescence was distributed along the intestinal epithelium, whereas the DSS group showed markedly weakened signals, indicating reduced protein expression. NaB, PDTC, or combined treatment restored green fluorescence intensity, suggesting recovered protein expression. Western blot analysis (**Figures 10D–G**) additionally validated marked decreases in Claudin-1, Occludin, and ZO-1 within the DSS group (*P* < 0.0001). Relative to the DSS group, NaB increased Claudin-1 (*P* < 0.01), Occludin (*P* < 0.0001), and ZO-1 (*P* < 0.01); PDTC increased Claudin-1 (*P* < 0.01), Occludin (*P* < 0.0001), and ZO-1 (*P* < 0.001); and combined treatment increased Claudin-1, Occludin, and ZO-1 (all *P* < 0.0001). These results report comparisons with the DSS group only and do not establish whether the combination differs from either monotherapy.

### 3.11 Colonic p-p65/p65 and p-IκBα/IκBα ratios after NaB, PDTC, and combined NaB/PDTC treatment in DSS-induced UC mice

**Figure 11:**
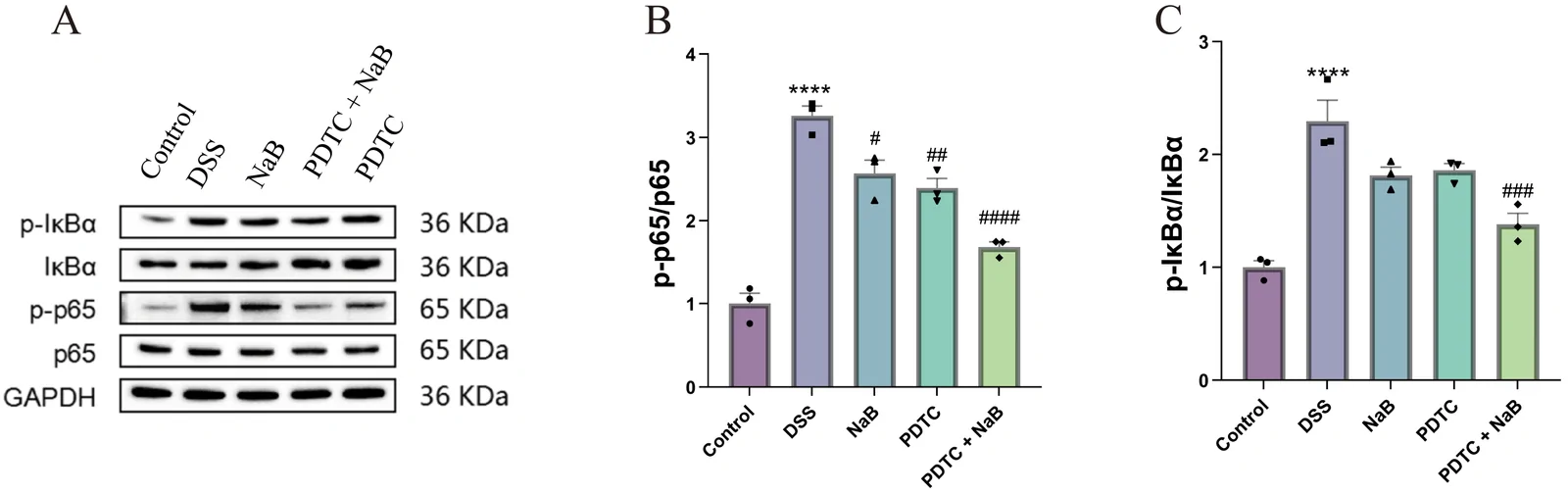
Colonic p-p65/p65 and p-IκBα/IκBα ratios after NaB, PDTC, and combined NaB/PDTC treatment in DSS-induced UC mice. **(A)** Representative Western blot bands of p-IκBα, IκBα, p-p65, and p65 in colonic tissues. **(B, C)** Quantification of the p-p65/p65 and p-IκBα/IκBα ratios. For **(B, C)**, n = 3 biologically independent samples per group. Data are presented as mean ± standard error of the mean (SEM). Group differences were analyzed using one-way ANOVA with Bonferroni correction or Dunnett’s T3 multiple-comparisons test when variances were unequal. ****P < 0.0001 versus the Control group; ^#^*P* < 0.05, ^##^*P* < 0.01, ^###^*P* < 0.001, and ^####^*P* < 0.0001 versus the DSS group. UC, ulcerative colitis.

As illustrated in **Figure 11**, Western blot analysis showed that DSS treatment markedly increased the p-p65/p65 and p-IκBα/IκBα ratios relative to the control group (both *P* < 0.0001). Compared with the DSS group, NaB and PDTC monotherapies significantly reduced the p-p65/p65 ratio (*P* < 0.05 and *P* < 0.01, respectively), whereas neither monotherapy significantly altered the p-IκBα/IκBα ratio. Combined NaB and PDTC treatment significantly reduced both the p-p65/p65 ratio (*P* < 0.0001) and the p-IκBα/IκBα ratio (*P* < 0.001). These comparisons were made against the DSS group; the combination was not directly compared with either monotherapy.

### 3.12 NaB and PDTC interventions modulate Th17/Treg proportions in the spleen and mesenteric lymph nodes of DSS-induced UC mice

**Figure 12:**
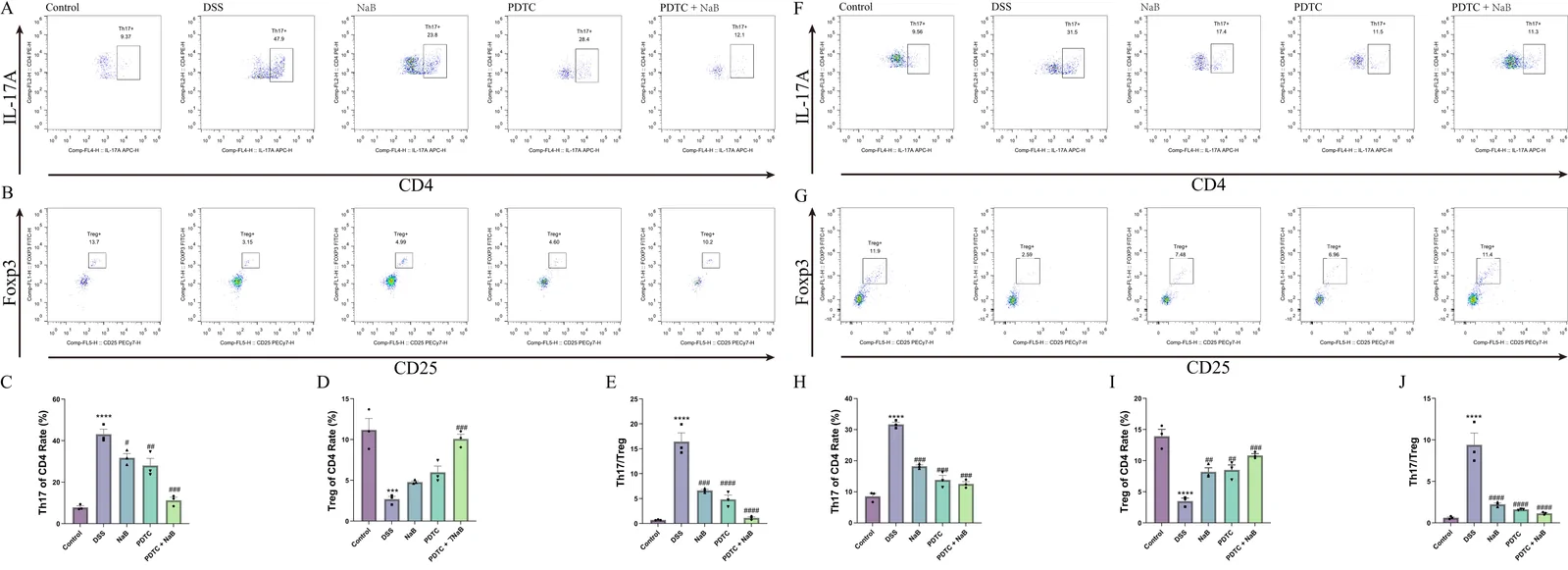
NaB and PDTC interventions modulate Th17/Treg proportions in the spleen and mesenteric lymph nodes of DSS-induced UC mice. **(A, B)** Representative flow cytometry plots of Th17 and Treg cells in spleen tissues. **(C–E)** Quantification of Th17 cells, Treg cells, and the Th17/Treg ratio. **(F, G)** Representative flow cytometry plots of Th17 and Treg cells in lymph nodes. **(H–J)** Quantification of Th17 cells, Treg cells, and the Th17/Treg ratio. Relative to the DSS group, NaB, PDTC, and combined NaB + PDTC treatments were associated with changes in selected Th17 and Treg proportions and Th17/Treg ratios in the spleen and mesenteric lymph nodes, as indicated in the corresponding panels. For **(C–E, H–J)**, n = 3 biologically independent samples per group. Data are presented as mean ± standard error of the mean (SEM). Group differences were analyzed using one-way ANOVA with Bonferroni correction or Dunnett’s T3 multiple-comparisons test when variances were unequal. ***P < 0.001 and ****P < 0.0001 versus the Control group; ^#^*P* < 0.05, ^##^*P* < 0.01, ^###^*P* < 0.001, and ^####^*P* < 0.0001 versus the DSS group. UC, ulcerative colitis.

As shown in **Figures 12A–E**, DSS-treated mice had significantly higher Th17 cell proportions and lower Treg cell proportions in splenic tissue than the control group, with a significantly increased Th17/Treg ratio (*P* < 0.0001, *P* < 0.001, and *P* < 0.0001, respectively; **Supplementary Figure 2** shows the gating strategy for flow-cytometric identification of Th17 and Treg cells in the NaB/PDTC study). Compared to the DSS group, NaB intervention markedly decreased Th17 cell proportions and the Th17/Treg ratio (both *P* < 0.001); PDTC intervention similarly reduced Th17 cell proportions (*P* < 0.001) and the Th17/Treg ratio (*P* < 0.0001). Relative to the DSS group, combined PDTC and NaB intervention elevated Treg cell proportions (*P* < 0.001) and diminished both Th17 cell proportions and the Th17/Treg ratio (both *P* < 0.001).

As shown in **Figures 12F–J**, DSS-treated mice also showed significantly higher Th17 cell proportions, lower Treg cell proportions, and an increased Th17/Treg ratio in lymph nodes versus the control group (all *P* < 0.0001). Relative to the DSS group, NaB and PDTC interventions significantly decreased Th17 cell proportions (both *P* < 0.001), increased Treg cell proportions (both *P* < 0.01), and reduced the Th17/Treg ratio (both *P* < 0.0001). Relative to the DSS group, combined PDTC and NaB intervention decreased Th17 cell proportions (*P* < 0.001), increased Treg cell proportions (*P* < 0.001), and reduced the Th17/Treg ratio (*P* < 0.0001).

### 3.13 NaB- and PDTC-associated exploratory trends in colonic microbiota richness and community structure in DSS-induced UC mice

**Figure 13:**
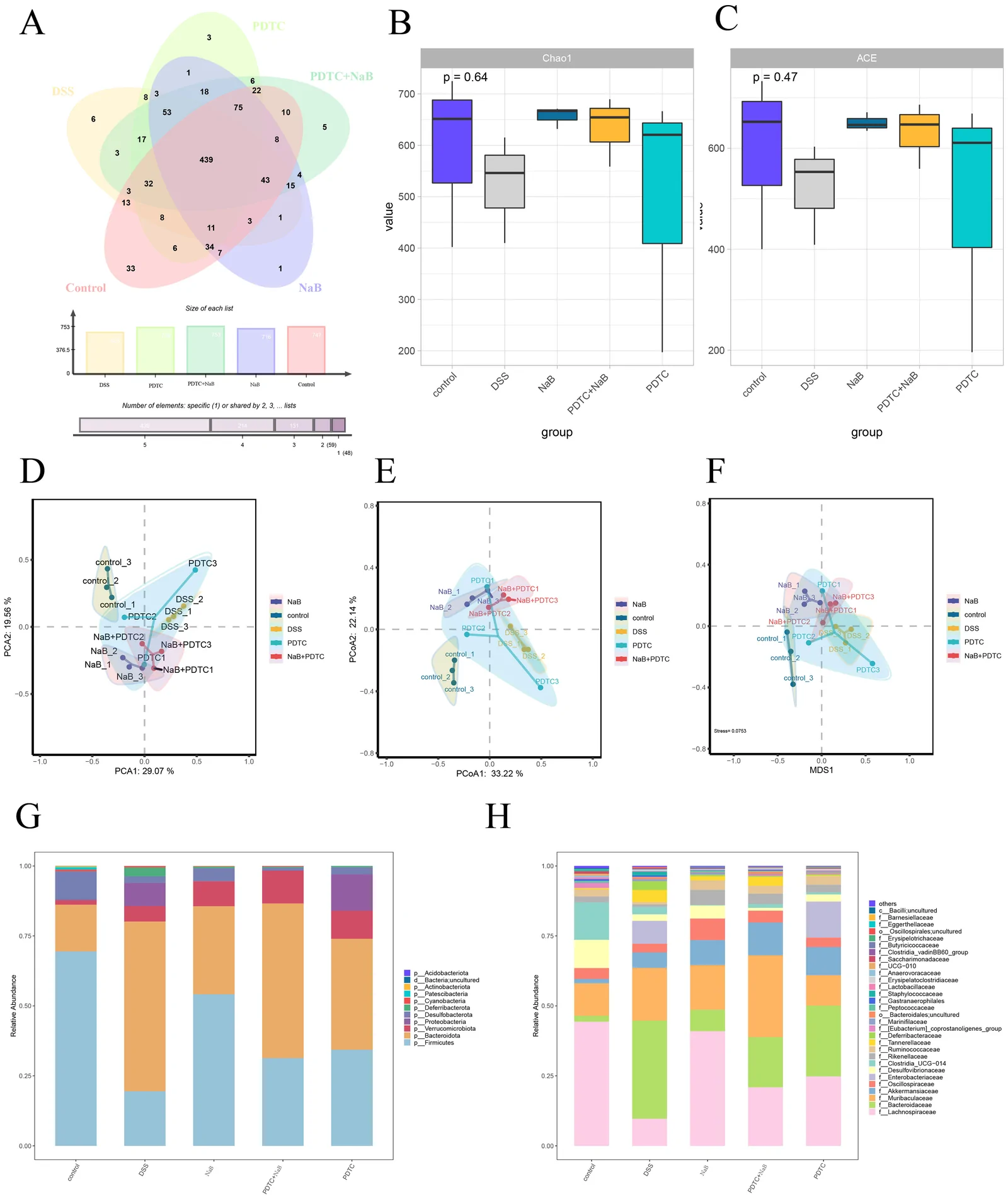
NaB- and PDTC-associated exploratory trends in colonic microbiota richness and community structure in DSS-induced UC mice. **(A)** Venn diagram showing OTU distribution and overlap among the Control, DSS, NaB, PDTC, and PDTC + NaB groups. **(B, C)** Alpha-diversity indices (Chao1 and ACE), with Kruskal–Wallis *P* values shown. **(D–F)** Beta-diversity analyses based on PCA, PCoA, and NMDS. **(G, H)** Relative abundances of colonic microbiota at the phylum and family levels. For **(A–H)**, n = 3 independent colonic-content samples per group. Panels **(B, C)** were analyzed using the Kruskal-Wallis test, with global *P* values shown; no multiple-testing correction was applied because no pairwise comparisons were performed. Group separation in **(D–F)** was assessed by PERMANOVA (Adonis). **(G, H)** show descriptive relative-abundance profiles without statistical annotations. UC, ulcerative colitis.

The Venn diagram of OTU distribution showed 747, 658, 716, 736, and 753 OTUs in the Control, DSS, NaB, PDTC, and PDTC+NaB groups, respectively; 439 OTUs were shared across all five groups. Microbiome analysis included n = 3 samples per group. The overlap between the control and intervention groups suggested a trend toward control-like microbial profiles (**Figure 13A**). Alpha-diversity analysis showed numerically lower ACE and Chao1 indices in the DSS group and numerically higher values after NaB, PDTC, and PDTC + NaB treatment; these patterns were interpreted as exploratory (**Figures 13B,C**).

Beta diversity analysis suggested treatment-associated differences in microbiota structure. Adonis/PERMANOVA showed significant overall group effects at the OTU level (R^2^ = 0.5219, F = 2.7287, P = 0.002), genus level (R^2^ = 0.5083, F = 2.5845, P = 0.014), and phylum level (R^2^ = 0.5727, F = 3.3503, P = 0.028). However, pairwise ANOSIM comparisons were not significant after BH correction; therefore, PCA, PCoA, and NMDS patterns were interpreted as community-level trends rather than definitive pairwise separation (**Figures 13D–F**).

Relative-abundance profiles suggested apparent DSS-associated increases in Bacteroidota and Proteobacteria and a decrease in Firmicutes (**Figure 13G**). At the family level, the profiles suggested apparent differences in Bacteroidaceae, Muribaculaceae, Enterobacteriaceae, Akkermansiaceae, and Lachnospiraceae (**Figure 13H**). Following intervention, these taxa showed trend-level shifts; however, the corresponding comparisons were not significant after multiple-testing correction. These taxon-level observations are therefore exploratory.

## 4 Discussion

This study showed that FMT attenuated DSS-induced colitis in mice, as reflected by reduced body-weight loss and DAI scores, increased colon length, and improved colonic histopathology. These phenotypic improvements were accompanied by lower pro-inflammatory cytokine levels, higher anti-inflammatory cytokine levels, increased expression of tight-junction proteins, reduced p65 phosphorylation, and shifts in splenic and mesenteric lymph-node Th17/Treg proportions. FMT was also associated with exploratory trends toward control-like microbial richness and community structure, together with changes in fecal SCFA profiles. In a separate intervention experiment, NaB, PDTC, and NaB plus PDTC were each associated with changes in selected outcomes relative to DSS. Because the combination was not directly compared with either monotherapy, the NaB/PDTC findings are interpreted descriptively. Together, these findings show parallel changes in fecal SCFA concentrations, the colonic p-p65/p65 ratio, and Th17/Treg proportions in the spleen and mesenteric lymph nodes; they do not establish causal relationships among these outcomes or link them sequentially to FMT efficacy.

Recent randomized controlled trials indicate that the clinical effects of FMT in UC vary substantially according to the treatment regimen and disease setting. In the LOTUS trial, antibiotic pretreatment followed by oral lyophilized FMT achieved corticosteroid-free clinical remission with endoscopic remission or response at week 8 in 8/15 patients (53%), compared with 3/20 (15%) patients receiving placebo (difference, 38.3 percentage points; *P* = 0.027) (21). In contrast, the CRAFT UC trial reported week-8 steroid-free clinical remission in 2/17 (11.8%) participants receiving standard single-donor FMT and 4/19 (21.1%) receiving diet-conditioned FMT, versus 6/15 (40%) receiving the UC Exclusion Diet alone, with no significant between-group differences (22). Likewise, in quiescent UC, a single-dose FMT regimen met the prespecified 12-month maintenance endpoint in 13/24 patients (54%), compared with 10/24 (41%) in the autologous-transplantation control group (*P* = 0.660) (23). These heterogeneous outcomes highlight the potential influence of donor selection, standardized preparation, dosing, delivery route, concomitant interventions, and recipient-specific factors. Long-term safety surveillance and standardized quality-control procedures remain essential barriers to clinical translation and limit direct extrapolation of short-term preclinical effect sizes to patients. These considerations also underscore the value of mechanistic studies examining immunoregulatory pathways associated with FMT response.

Previous studies have shown that abnormal changes in cytokines, including IL-10, IL-17A, IL-21, IL-22, and TGF-β1, are closely linked to inflammatory responses and immune dysregulation in UC and other immune-related diseases. These cytokines may also serve as useful indicators for assessing disease activity and immunoregulatory status (24, 25). This study found that FMT decreased colonic IL-17A and IL-21 expression while enhancing TGF-β1 release in UC mice. Meanwhile, serum IL-21 levels were reduced, whereas IL-10 and TGF-β1 levels were increased, indicating that FMT can restore the balance between pro-inflammatory and anti-inflammatory cytokines. IL-17A is a representative effector cytokine of Th17 cells that promotes pro-inflammatory mediator release by activating inflammatory signaling pathways, thereby aggravating intestinal mucosal inflammatory injury. Its expression is positively associated with UC disease activity (24, 26). IL-21, predominantly produced by activated CD4^+^ T cells, promotes Th17 cell differentiation, amplifies inflammatory responses, and induces IL-22 production. Sustained IL-21 overexpression is closely associated with chronic intestinal inflammation in IBD (27–29). In this study, FMT decreased IL-21 levels in both colon and serum, suggesting that its protective effects may be partly mediated by suppression of the Th17-associated inflammatory network. In contrast, TGF-β1 and IL-10 are key anti-inflammatory factors required for intestinal immune homeostasis. TGF-β1 promotes Treg cell differentiation, preserves mucosal barrier integrity, and suppresses excessive inflammation, and abnormal TGF-β1 signaling has been linked to sustained inflammation in UC (27, 30). IL-10 exerts negative immunoregulatory effects mainly by inhibiting macrophage-, dendritic cell-, and Th17-associated inflammatory responses (31). Therefore, increased TGF-β1 and IL-10 expression after FMT suggests that FMT may improve the intestinal inflammatory microenvironment in UC mice by strengthening Treg-associated immunoregulatory functions and restoring the balance between pro-inflammatory and anti-inflammatory mediators. Collectively, FMT-mediated cytokine network modulation may represent an important mechanism underlying its therapeutic effects in UC.

16S rRNA sequencing indicated that, after FMT intervention, α-diversity indices and β-diversity ordination patterns showed exploratory trends toward the control profile. Relative-abundance profiles also suggested apparent FMT-associated shifts in *Bacteroidota* and *Firmicutes* at the phylum level and in *Lachnospiraceae*, *Bacteroidaceae*, *Muribaculaceae*, and *Enterobacteriaceae* at the family level. Given the small sample size and the lack of significant genus-level and pairwise ANOSIM results after multiple-testing correction, these patterns should be interpreted as exploratory rather than as evidence that specific taxa were corrected, restored, or recovered. *Bacteroidota* and *Firmicutes* are the two dominant phyla required for intestinal homeostasis, and their imbalance is closely linked to UC onset and progression. *Bacteroidota* contributes to complex carbohydrate degradation and SCFAs production, and reduced *Bacteroidota* abundance has been associated with impaired barrier function and aggravated inflammation (5, 32). Additionally, certain *Bacteroidota* members promote Treg responses and inhibit IL-17 production through polysaccharide A, thereby alleviating colonic inflammation (33). Firmicutes is a diverse phylum whose relative abundance was reduced in DSS-induced colitis and partially restored after FMT. Butyrate serves as a key energy source for colonocytes and also inhibits NF-κB signaling activation and reduces inflammatory responses (34, 35). Moreover, at the family level, Lachnospiraceae abundance has been reported to be inversely associated with UC disease activity and barrier injury (36–39), while *Muribaculaceae* supports mucus barrier maintenance, SCFAs production, and colonization resistance against pathogens (40). In contrast, *Enterobacteriaceae* is enriched in pro-inflammatory components such as lipopolysaccharide, which can amplify inflammatory responses through pathways including TLR4 activation and thereby promote UC progression (41). Collectively, FMT was associated with partial recovery of microbial richness and community structure and with trend-level shifts in several bacterial taxa. These ecological changes occurred alongside disease improvement. However, because most taxa-level differences were exploratory after multiple-testing correction and no taxon-butyrate correlation or functional microbial perturbation was performed, the present data do not establish that changes in particular taxa caused the alterations in fecal butyrate or the protective phenotype.

Western blot analysis showed that FMT-treated mice had a significantly lower colonic p-p65/p65 ratio than DSS-treated mice, whereas the p-IκBα/IκBα ratio did not differ significantly between the two groups. This finding is consistent with evidence from another DSS-induced chronic-colitis model, in which a prebiotic intervention was associated with attenuation of colitis, reduced phosphorylation of IKKα/β, IκBα, and p65, and concomitant changes in gut microbiota and SCFA concentrations (42). Subsequent flow cytometric analysis revealed that FMT reduced Th17 proportions, increased Treg proportions, and lowered the Th17/Treg ratio in the spleen and mesenteric lymph nodes relative to DSS. NF-κB serves as a crucial transcription factor in inflammatory responses and acts as a significant regulator of T-cell differentiation. Abnormal NF-κB activation promotes Th17 cell differentiation, amplifies inflammatory responses, and suppresses Treg cell-mediated immune tolerance (43–45). Viewed in this biological context, the reduced colonic p-p65/p65 ratio and tissue-specific Th17/Treg changes define a focused set of host responses associated with FMT. Together with the observed cytokine changes, increased colonic tight-junction protein expression, exploratory microbiota patterns, and higher endpoint fecal butyrate concentration, these findings provide an integrated, tissue-resolved characterization of FMT-associated disease improvement. This multi-domain profile identifies specific, testable relationships for future studies designed to determine how microbial and host responses are connected. Because only colonic phosphorylation ratios and Th17/Treg proportions in the spleen and mesenteric lymph nodes were assessed, these data do not establish NF-κB pathway activity, causal ordering, or local colonic T-cell changes. The separate NaB/PDTC experiment identified selected outcomes that changed relative to DSS after NaB, PDTC, or combined treatment. Although no direct comparisons were made between the combination and either monotherapy, these findings help prioritize specific biochemical and immune measures for future factorial studies.

Targeted metabolomic analysis showed that endpoint fecal butyrate concentration was higher in the FMT group than in both the DSS and 5-ASA groups. These group differences identify endpoint fecal butyrate concentration as a candidate feature of FMT-associated disease improvement and provide a focused basis for longitudinal and functional validation. However, the single endpoint measurement does not establish butyrate production, metabolic flux, absorption, or restoration to baseline. In DSS-induced UC mice, NaB treatment significantly decreased serum IL-21 levels, increased TGF-β1 levels, and reduced the colonic p-p65/p65 ratio relative to DSS. These measured changes occurred alongside NaB-related disease improvement. Previous studies have also reported well-characterized anti-inflammatory and immunomodulatory properties of butyrate (46, 47). On one hand, butyrate suppresses IL-21 expression through epigenetic regulation (48). On the other hand, it promotes TGF-β1 and IL-10 secretion and induces tolerogenic antigen-presenting cell formation (49, 50), thereby helping restore the pro-inflammatory/anti-inflammatory cytokine balance. Beyond immune modulation, butyrate also provides marked protection for the intestinal mucosal barrier. In this study, NaB treatment increased the expression of Claudin-1, Occludin, and ZO-1 that had been reduced by DSS exposure, a pattern consistent with improved epithelial tight-junction integrity. This result is consistent with previous reports showing that butyrate stabilizes membrane localization and restores barrier function by regulating tight-junction protein transcription (51–53). Moreover, NaB intervention markedly improved the Th17/Treg imbalance in the spleen and lymph nodes. This immune rebalancing occurred alongside reduced p65 phosphorylation, supporting a biologically coherent relationship between NF-κB-related signaling and Th17/Treg homeostasis in NaB-treated mice (54–57). Additionally, NaB treatment was associated with greater microbial diversity and recovery of beneficial taxa, including Firmicutes and Lachnospiraceae. These coordinated changes provide a plausible biological context for reciprocal microbiota–metabolite interactions during intestinal microecological recovery (58–61). Collectively, the FMT experiment and the separate NaB/PDTC experiment provide a multidimensional, tissue-resolved profile of treatment-associated disease improvement. Across these experiments, improvement coincided with parallel changes in endpoint fecal butyrate concentration, the colonic p-p65/p65 ratio, tight-junction protein expression, and splenic and mesenteric lymph node Th17/Treg proportions. These findings identify testable relationships and provide an empirical basis for future mechanistic studies, but do not establish a mechanistic network or causality. Because the NaB/PDTC combination was not directly compared with either monotherapy, no conclusions about combined effects can be drawn.

This study identified parallel associations among FMT-related improvement, exploratory microbiota patterns, endpoint fecal butyrate concentration, the colonic p-p65/p65 ratio, and Th17/Treg proportions in the spleen and mesenteric lymph nodes; however, several limitations remain. First, the DSS-induced mouse model does not fully reflect the chronic course, immune heterogeneity, or individualized microbiota of human UC. Second, only male BALB/c mice were used, and potential sex-related differences in immune responses, microbiota composition, and DSS susceptibility require consideration. Third, the DSS group lacked a vehicle gavage control matched to the FMT and 5-ASA groups; therefore, effects of repeated gavage, saline exposure, handling stress, and the bacterial suspension matrix cannot be excluded. Fourth, Th17 and Treg cells were assessed only in the spleen and mesenteric lymph nodes, not in the colonic lamina propria; therefore, these findings do not establish local colonic T-cell rebalancing. No fixable viability dye was used, preventing reliable exclusion of nonviable cells generated during tissue dissociation or PMA/ionomycin stimulation. Although FSC/SSC gating reduced debris, it did not provide viability discrimination and may have affected the estimated Th17 and Treg frequencies. Fifth, microbiome analyses were limited to three samples per group. Genus-level and pairwise ANOSIM comparisons were non-significant after multiple-testing correction; thus, community- and taxon-level patterns are exploratory. Potential cage effects were not assessed. Sixth, because PDTC may have off-target effects, the NaB/PDTC findings support biological plausibility rather than pathway-specific causality. Seventh, the key effector microbes, functional metabolites, and causal relationships among the FMT-associated changes remain undefined. As correlations between specific taxa and fecal butyrate concentrations were not examined, these changes were interpreted as parallel associations. Eighth, the FMT donor source, preparation, intervention duration, and administration regimen were limited. The FMT preparation also lacked comprehensive quality control, including bacterial-load standardization, viability assessment, and profiling of the donor and final preparations, potentially affecting procedural reproducibility and interpretation of microbiome-related outcomes. Future studies integrating multi-omics analyses, functional validation, optimized controls, fixation-compatible viability staining, and standardized FMT quality control should clarify these relationships and support clinical translation.

## 5 Conclusion

In conclusion, FMT alleviated colonic injury and clinical symptoms in DSS-induced UC mice. This improvement was accompanied by altered cytokine concentrations, increased colonic tight-junction protein expression, exploratory microbiota shifts, and increased endpoint fecal butyrate concentration. It also coincided with a reduced colonic p-p65/p65 ratio and altered Th17/Treg proportions in the spleen and mesenteric lymph nodes. Together, these findings provide an integrated, tissue-resolved characterization of FMT-associated disease improvement and identify specific relationships for future mechanistic testing. In a separate experiment, NaB, PDTC, and their combination altered selected outcomes relative to DSS, with the combination reducing both the colonic p-p65/p65 and p-IκBα/IκBα ratios. Because the combination was not directly compared with either monotherapy, no conclusions about combined effects can be drawn. The observed associations do not establish causality or changes in Th17/Treg proportions within the colonic mucosa.

## Data Availability

The 16S rRNA gene sequencing data generated in this study have been deposited in the NCBI Sequence Read Archive under BioProject accession number PRJNA1468102 and are publicly available at https://www.ncbi.nlm.nih.gov/sra/PRJNA1468102. Other data supporting the findings of this study are available from the corresponding author upon reasonable request.
