# Supplementary material for: Fecal microbiota transplantation alleviates DSS-induced colitis: increased fecal butyrate, reduced colonic p65 phosphorylation, and altered Th17/Treg ratios in the spleen and mesenteric lymph nodes
Source: Front Immunol. 2026 Aug 17;17:1903198. doi: 10.3389/fimmu.2026.1903198 (PMC13527040; doi:10.3389/fimmu.2026.1903198)
Supplement: Supplementary file 1 [file Table1.docx]

**Supplementary File: Full-length Original Western Blot Images**

**Scope:** This file provides full-length original Western blot exposure images corresponding to the cropped Western blot panels in the manuscript. Source images were taken from the original TIFF exposure folders for animal experiment 1 and animal experiment 2.

**Image handling:** The original TIFF images were converted to PNG solely for Word compatibility. No cropping, splicing, brightness/contrast adjustment, or band enhancement was applied. Red boxes are annotations added only to mark visible membrane/exposure boundaries while retaining the full original image canvas.

**Labelling:** Each image is labelled with the manuscript figure panel, target protein/loading control, treatment groups, lane-order descriptor, original file name, and exposure identifier. Molecular-weight ladder/marker lanes are retained when visible in the original exposure image.

# Figure-to-Source Mapping

| **Manuscript panel** | **Experiment** | **Targets/loading control** | **Treatment groups** | **Original source folder** |
| --- | --- | --- | --- | --- |
| Figure 3D | Animal experiment 1 | Claudin-1, Occludin, ZO-1; GAPDH loading control | Normal, DSS, FMT, 5-ASA | Animal experiment 1, WB original exposure TIFF folder |
| Figure 4A | Animal experiment 1 | p-IKBalpha (p-IκBα), IKBalpha (IκBα), p-p65, p65; GAPDH loading control | Normal, DSS, FMT, 5-ASA | Animal experiment 1, WB original exposure TIFF folder |
| Figure 10D | Animal experiment 2 | Claudin-1, Occludin, ZO-1; GAPDH loading control | Control, DSS, NaB, PDTC, PDTC + NaB | Animal experiment 2, WB original exposure TIFF folder, tight-junction subset |
| Figure 11A | Animal experiment 2 | p-IKBalpha (p-IκBα), IKBalpha (IκBα), p-p65, p65; GAPDH loading control | Control, DSS, NaB, PDTC, PDTC + NaB | Animal experiment 2, WB original exposure TIFF folder, NF-kappaB subset |

# Figure 3D: Animal experiment 1

**1. Figure 3D - Claudin-1 (exposure 1). Groups: Normal, DSS, FMT, 5-ASA. Lane order: Normal, DSS, FMT, and 5-ASA groups as arranged in the original exposure image. Original file: Claudin 1.tiff. Source folder: Animal experiment 1, WB original exposure TIFF folder.** Note: Original file uses the name 'Claudin'; mapped to manuscript target Claudin-1.

**2. Figure 3D - Claudin-1 (exposure 1-2). Groups: Normal, DSS, FMT, 5-ASA. Lane order: Normal, DSS, FMT, and 5-ASA groups as arranged in the original exposure image. Original file: Claudin 1-2.tiff. Source folder: Animal experiment 1, WB original exposure TIFF folder.** Note: Original file uses the name 'Claudin'; mapped to manuscript target Claudin-1.

**3. Figure 3D - Occludin (exposure 1). Groups: Normal, DSS, FMT, 5-ASA. Lane order: Normal, DSS, FMT, and 5-ASA groups as arranged in the original exposure image. Original file: Occludin 1.tiff. Source folder: Animal experiment 1, WB original exposure TIFF folder.**

**4. Figure 3D - Occludin (exposure 1-2). Groups: Normal, DSS, FMT, 5-ASA. Lane order: Normal, DSS, FMT, and 5-ASA groups as arranged in the original exposure image. Original file: Occludin 1-2.tiff. Source folder: Animal experiment 1, WB original exposure TIFF folder.**

**5. Figure 3D - ZO-1 (exposure 1). Groups: Normal, DSS, FMT, 5-ASA. Lane order: Normal, DSS, FMT, and 5-ASA groups as arranged in the original exposure image. Original file: ZO-1 1.tiff. Source folder: Animal experiment 1, WB original exposure TIFF folder.**

**6. Figure 3D - ZO-1 (exposure 1-2). Groups: Normal, DSS, FMT, 5-ASA. Lane order: Normal, DSS, FMT, and 5-ASA groups as arranged in the original exposure image. Original file: ZO-1 1-2.tiff. Source folder: Animal experiment 1, WB original exposure TIFF folder.**

**7. Figure 3D - GAPDH (exposure 1). Groups: Normal, DSS, FMT, 5-ASA. Lane order: Normal, DSS, FMT, and 5-ASA groups as arranged in the original exposure image. Original file: GAPDH 1.tiff. Source folder: Animal experiment 1, WB original exposure TIFF folder.** Note: Loading control for Figure 3D.

**8. Figure 3D - GAPDH (exposure 1-2). Groups: Normal, DSS, FMT, 5-ASA. Lane order: Normal, DSS, FMT, and 5-ASA groups as arranged in the original exposure image. Original file: GAPDH 1-2.tiff. Source folder: Animal experiment 1, WB original exposure TIFF folder.** Note: Loading control for Figure 3D.

# Figure 4A: Animal experiment 1

**9. Figure 4A - p-IKBalpha (p-IκBα) (exposure 1). Groups: Normal, DSS, FMT, 5-ASA. Lane order: Normal, DSS, FMT, and 5-ASA groups as arranged in the original exposure image. Original file: p-IKBa 1.tiff. Source folder: Animal experiment 1, WB original exposure TIFF folder.**

**10. Figure 4A - p-IKBalpha (p-IκBα) (exposure 1-2). Groups: Normal, DSS, FMT, 5-ASA. Lane order: Normal, DSS, FMT, and 5-ASA groups as arranged in the original exposure image. Original file: p-IKBa 1-2.tiff. Source folder: Animal experiment 1, WB original exposure TIFF folder.**

**11. Figure 4A - IKBalpha (IκBα) (exposure 1). Groups: Normal, DSS, FMT, 5-ASA. Lane order: Normal, DSS, FMT, and 5-ASA groups as arranged in the original exposure image. Original file: IKBa 1.tiff. Source folder: Animal experiment 1, WB original exposure TIFF folder.**

**12. Figure 4A - IKBalpha (IκBα) (exposure 1-2). Groups: Normal, DSS, FMT, 5-ASA. Lane order: Normal, DSS, FMT, and 5-ASA groups as arranged in the original exposure image. Original file: IKBa 1-2.tiff. Source folder: Animal experiment 1, WB original exposure TIFF folder.**

**13. Figure 4A - p-p65 (exposure 1). Groups: Normal, DSS, FMT, 5-ASA. Lane order: Normal, DSS, FMT, and 5-ASA groups as arranged in the original exposure image. Original file: p-p65 1.tiff. Source folder: Animal experiment 1, WB original exposure TIFF folder.**

**14. Figure 4A - p-p65 (exposure 1-2). Groups: Normal, DSS, FMT, 5-ASA. Lane order: Normal, DSS, FMT, and 5-ASA groups as arranged in the original exposure image. Original file: p-p65 1-2.tiff. Source folder: Animal experiment 1, WB original exposure TIFF folder.**

**15. Figure 4A - p65 (exposure 1). Groups: Normal, DSS, FMT, 5-ASA. Lane order: Normal, DSS, FMT, and 5-ASA groups as arranged in the original exposure image. Original file: p65 1.tiff. Source folder: Animal experiment 1, WB original exposure TIFF folder.**

**16. Figure 4A - p65 (exposure 1-2). Groups: Normal, DSS, FMT, 5-ASA. Lane order: Normal, DSS, FMT, and 5-ASA groups as arranged in the original exposure image. Original file: p65 1-2.tiff. Source folder: Animal experiment 1, WB original exposure TIFF folder.**

**17. Figure 4A - GAPDH (exposure 1). Groups: Normal, DSS, FMT, 5-ASA. Lane order: Normal, DSS, FMT, and 5-ASA groups as arranged in the original exposure image. Original file: GAPDH 2.tiff. Source folder: Animal experiment 1, WB original exposure TIFF folder.** Note: Loading control for Figure 4A.

**18. Figure 4A - GAPDH (exposure 1-2). Groups: Normal, DSS, FMT, 5-ASA. Lane order: Normal, DSS, FMT, and 5-ASA groups as arranged in the original exposure image. Original file: GAPDH 2-2.tiff. Source folder: Animal experiment 1, WB original exposure TIFF folder.** Note: Loading control for Figure 4A.

# Figure 10D: Animal experiment 2

**19. Figure 10D - Claudin-1 (exposure 1). Groups: Control, DSS, NaB, PDTC, PDTC + NaB. Lane order: Control, DSS, NaB, PDTC, and PDTC + NaB groups as arranged in the original exposure image. Original file: Claudin-1 1.tiff. Source folder: Animal experiment 2, WB original exposure TIFF folder, tight-junction subset.**

**20. Figure 10D - Claudin-1 (exposure 1-2). Groups: Control, DSS, NaB, PDTC, PDTC + NaB. Lane order: Control, DSS, NaB, PDTC, and PDTC + NaB groups as arranged in the original exposure image. Original file: Claudin-1 1-2.tiff. Source folder: Animal experiment 2, WB original exposure TIFF folder, tight-junction subset.**

**21. Figure 10D - Claudin-1 (exposure 2). Groups: Control, DSS, NaB, PDTC, PDTC + NaB. Lane order: Control, DSS, NaB, PDTC, and PDTC + NaB groups as arranged in the original exposure image. Original file: Claudin-1 2.tiff. Source folder: Animal experiment 2, WB original exposure TIFF folder, tight-junction subset.**

**22. Figure 10D - Claudin-1 (exposure 2-2). Groups: Control, DSS, NaB, PDTC, PDTC + NaB. Lane order: Control, DSS, NaB, PDTC, and PDTC + NaB groups as arranged in the original exposure image. Original file: Claudin-1 2-2.tiff. Source folder: Animal experiment 2, WB original exposure TIFF folder, tight-junction subset.**

**23. Figure 10D - Occludin (exposure 1). Groups: Control, DSS, NaB, PDTC, PDTC + NaB. Lane order: Control, DSS, NaB, PDTC, and PDTC + NaB groups as arranged in the original exposure image. Original file: Occludin 1.tiff. Source folder: Animal experiment 2, WB original exposure TIFF folder, tight-junction subset.**

**24. Figure 10D - Occludin (exposure 1-2). Groups: Control, DSS, NaB, PDTC, PDTC + NaB. Lane order: Control, DSS, NaB, PDTC, and PDTC + NaB groups as arranged in the original exposure image. Original file: Occludin 1-2.tiff. Source folder: Animal experiment 2, WB original exposure TIFF folder, tight-junction subset.**

**25. Figure 10D - Occludin (exposure 2). Groups: Control, DSS, NaB, PDTC, PDTC + NaB. Lane order: Control, DSS, NaB, PDTC, and PDTC + NaB groups as arranged in the original exposure image. Original file: Occludin 2.tiff. Source folder: Animal experiment 2, WB original exposure TIFF folder, tight-junction subset.**

**26. Figure 10D - Occludin (exposure 2-2). Groups: Control, DSS, NaB, PDTC, PDTC + NaB. Lane order: Control, DSS, NaB, PDTC, and PDTC + NaB groups as arranged in the original exposure image. Original file: Occludin 2-2.tiff. Source folder: Animal experiment 2, WB original exposure TIFF folder, tight-junction subset.**

**27. Figure 10D - ZO-1 (exposure 1). Groups: Control, DSS, NaB, PDTC, PDTC + NaB. Lane order: Control, DSS, NaB, PDTC, and PDTC + NaB groups as arranged in the original exposure image. Original file: ZO-1 1.tiff. Source folder: Animal experiment 2, WB original exposure TIFF folder, tight-junction subset.**

**28. Figure 10D - ZO-1 (exposure 1-2). Groups: Control, DSS, NaB, PDTC, PDTC + NaB. Lane order: Control, DSS, NaB, PDTC, and PDTC + NaB groups as arranged in the original exposure image. Original file: ZO-1 1-2.tiff. Source folder: Animal experiment 2, WB original exposure TIFF folder, tight-junction subset.**

**29. Figure 10D - ZO-1 (exposure 2). Groups: Control, DSS, NaB, PDTC, PDTC + NaB. Lane order: Control, DSS, NaB, PDTC, and PDTC + NaB groups as arranged in the original exposure image. Original file: ZO-1 2.tiff. Source folder: Animal experiment 2, WB original exposure TIFF folder, tight-junction subset.**

**30. Figure 10D - ZO-1 (exposure 2-2). Groups: Control, DSS, NaB, PDTC, PDTC + NaB. Lane order: Control, DSS, NaB, PDTC, and PDTC + NaB groups as arranged in the original exposure image. Original file: ZO-1 2-2.tiff. Source folder: Animal experiment 2, WB original exposure TIFF folder, tight-junction subset.**

**31. Figure 10D - GAPDH (exposure 1). Groups: Control, DSS, NaB, PDTC, PDTC + NaB. Lane order: Control, DSS, NaB, PDTC, and PDTC + NaB groups as arranged in the original exposure image. Original file: GAPDH 1.tiff. Source folder: Animal experiment 2, WB original exposure TIFF folder, tight-junction subset.** Note: Loading control for Figure 10D.

**32. Figure 10D - GAPDH (exposure 1-2). Groups: Control, DSS, NaB, PDTC, PDTC + NaB. Lane order: Control, DSS, NaB, PDTC, and PDTC + NaB groups as arranged in the original exposure image. Original file: GAPDH 1-2.tiff. Source folder: Animal experiment 2, WB original exposure TIFF folder, tight-junction subset.** Note: Loading control for Figure 10D.

**33. Figure 10D - GAPDH (exposure 2). Groups: Control, DSS, NaB, PDTC, PDTC + NaB. Lane order: Control, DSS, NaB, PDTC, and PDTC + NaB groups as arranged in the original exposure image. Original file: GAPDH 2.tiff. Source folder: Animal experiment 2, WB original exposure TIFF folder, tight-junction subset.** Note: Loading control for Figure 10D.

**34. Figure 10D - GAPDH (exposure 2-2). Groups: Control, DSS, NaB, PDTC, PDTC + NaB. Lane order: Control, DSS, NaB, PDTC, and PDTC + NaB groups as arranged in the original exposure image. Original file: GAPDH 2-2.tiff. Source folder: Animal experiment 2, WB original exposure TIFF folder, tight-junction subset.** Note: Loading control for Figure 10D.

# Figure 11A: Animal experiment 2

**35. Figure 11A - p-IKBalpha (p-IκBα) (exposure 1). Groups: Control, DSS, NaB, PDTC, PDTC + NaB. Lane order: Control, DSS, NaB, PDTC, and PDTC + NaB groups as arranged in the original exposure image. Original file: p-IKBa 1.tiff. Source folder: Animal experiment 2, WB original exposure TIFF folder, NF-kappaB subset.**

**36. Figure 11A - p-IKBalpha (p-IκBα) (exposure 1-2). Groups: Control, DSS, NaB, PDTC, PDTC + NaB. Lane order: Control, DSS, NaB, PDTC, and PDTC + NaB groups as arranged in the original exposure image. Original file: p-IKBa 1-2.tiff. Source folder: Animal experiment 2, WB original exposure TIFF folder, NF-kappaB subset.**

**37. Figure 11A - p-IKBalpha (p-IκBα) (exposure 2). Groups: Control, DSS, NaB, PDTC, PDTC + NaB. Lane order: Control, DSS, NaB, PDTC, and PDTC + NaB groups as arranged in the original exposure image. Original file: p-IKBa 2.tiff. Source folder: Animal experiment 2, WB original exposure TIFF folder, NF-kappaB subset.**

**38. Figure 11A - p-IKBalpha (p-IκBα) (exposure 2-2). Groups: Control, DSS, NaB, PDTC, PDTC + NaB. Lane order: Control, DSS, NaB, PDTC, and PDTC + NaB groups as arranged in the original exposure image. Original file: p-IKBa 2-2.tiff. Source folder: Animal experiment 2, WB original exposure TIFF folder, NF-kappaB subset.**

**39. Figure 11A - IKBalpha (IκBα) (exposure 1). Groups: Control, DSS, NaB, PDTC, PDTC + NaB. Lane order: Control, DSS, NaB, PDTC, and PDTC + NaB groups as arranged in the original exposure image. Original file: IKBa 1.tiff. Source folder: Animal experiment 2, WB original exposure TIFF folder, NF-kappaB subset.**

**40. Figure 11A - IKBalpha (IκBα) (exposure 1-2). Groups: Control, DSS, NaB, PDTC, PDTC + NaB. Lane order: Control, DSS, NaB, PDTC, and PDTC + NaB groups as arranged in the original exposure image. Original file: IKBa 1-2.tiff. Source folder: Animal experiment 2, WB original exposure TIFF folder, NF-kappaB subset.**

**41. Figure 11A - IKBalpha (IκBα) (exposure 2). Groups: Control, DSS, NaB, PDTC, PDTC + NaB. Lane order: Control, DSS, NaB, PDTC, and PDTC + NaB groups as arranged in the original exposure image. Original file: IKBa 2.tiff. Source folder: Animal experiment 2, WB original exposure TIFF folder, NF-kappaB subset.**

**42. Figure 11A - IKBalpha (IκBα) (exposure 2-2). Groups: Control, DSS, NaB, PDTC, PDTC + NaB. Lane order: Control, DSS, NaB, PDTC, and PDTC + NaB groups as arranged in the original exposure image. Original file: IKBa 2-2.tiff. Source folder: Animal experiment 2, WB original exposure TIFF folder, NF-kappaB subset.**

**43. Figure 11A - p-p65 (exposure 1). Groups: Control, DSS, NaB, PDTC, PDTC + NaB. Lane order: Control, DSS, NaB, PDTC, and PDTC + NaB groups as arranged in the original exposure image. Original file: p-p65 1.tiff. Source folder: Animal experiment 2, WB original exposure TIFF folder, NF-kappaB subset.**

**44. Figure 11A - p-p65 (exposure 1-2). Groups: Control, DSS, NaB, PDTC, PDTC + NaB. Lane order: Control, DSS, NaB, PDTC, and PDTC + NaB groups as arranged in the original exposure image. Original file: p-p65 1-2.tiff. Source folder: Animal experiment 2, WB original exposure TIFF folder, NF-kappaB subset.**

**45. Figure 11A - p-p65 (exposure 2). Groups: Control, DSS, NaB, PDTC, PDTC + NaB. Lane order: Control, DSS, NaB, PDTC, and PDTC + NaB groups as arranged in the original exposure image. Original file: p-p65 2.tiff. Source folder: Animal experiment 2, WB original exposure TIFF folder, NF-kappaB subset.**

**46. Figure 11A - p-p65 (exposure 2-2). Groups: Control, DSS, NaB, PDTC, PDTC + NaB. Lane order: Control, DSS, NaB, PDTC, and PDTC + NaB groups as arranged in the original exposure image. Original file: p-p65 2-2.tiff. Source folder: Animal experiment 2, WB original exposure TIFF folder, NF-kappaB subset.**

**47. Figure 11A - p65 (exposure 1). Groups: Control, DSS, NaB, PDTC, PDTC + NaB. Lane order: Control, DSS, NaB, PDTC, and PDTC + NaB groups as arranged in the original exposure image. Original file: p65 1.tiff. Source folder: Animal experiment 2, WB original exposure TIFF folder, NF-kappaB subset.**

**48. Figure 11A - p65 (exposure 1-2). Groups: Control, DSS, NaB, PDTC, PDTC + NaB. Lane order: Control, DSS, NaB, PDTC, and PDTC + NaB groups as arranged in the original exposure image. Original file: p65 1-2.tiff. Source folder: Animal experiment 2, WB original exposure TIFF folder, NF-kappaB subset.**

**49. Figure 11A - p65 (exposure 2). Groups: Control, DSS, NaB, PDTC, PDTC + NaB. Lane order: Control, DSS, NaB, PDTC, and PDTC + NaB groups as arranged in the original exposure image. Original file: p65 2.tiff. Source folder: Animal experiment 2, WB original exposure TIFF folder, NF-kappaB subset.**

**50. Figure 11A - p65 (exposure 2-2). Groups: Control, DSS, NaB, PDTC, PDTC + NaB. Lane order: Control, DSS, NaB, PDTC, and PDTC + NaB groups as arranged in the original exposure image. Original file: p65 2-2.tiff. Source folder: Animal experiment 2, WB original exposure TIFF folder, NF-kappaB subset.**

**51. Figure 11A - GAPDH (exposure 1). Groups: Control, DSS, NaB, PDTC, PDTC + NaB. Lane order: Control, DSS, NaB, PDTC, and PDTC + NaB groups as arranged in the original exposure image. Original file: GAPDH 1.tiff. Source folder: Animal experiment 2, WB original exposure TIFF folder, NF-kappaB subset.** Note: Loading control for Figure 11A.

**52. Figure 11A - GAPDH (exposure 1-2). Groups: Control, DSS, NaB, PDTC, PDTC + NaB. Lane order: Control, DSS, NaB, PDTC, and PDTC + NaB groups as arranged in the original exposure image. Original file: GAPDH 1-2.tiff. Source folder: Animal experiment 2, WB original exposure TIFF folder, NF-kappaB subset.** Note: Loading control for Figure 11A.

**53. Figure 11A - GAPDH (exposure 2). Groups: Control, DSS, NaB, PDTC, PDTC + NaB. Lane order: Control, DSS, NaB, PDTC, and PDTC + NaB groups as arranged in the original exposure image. Original file: GAPDH 2.tiff. Source folder: Animal experiment 2, WB original exposure TIFF folder, NF-kappaB subset.** Note: Loading control for Figure 11A.

**54. Figure 11A - GAPDH (exposure 2-2). Groups: Control, DSS, NaB, PDTC, PDTC + NaB. Lane order: Control, DSS, NaB, PDTC, and PDTC + NaB groups as arranged in the original exposure image. Original file: GAPDH 2-2.tiff. Source folder: Animal experiment 2, WB original exposure TIFF folder, NF-kappaB subset.** Note: Loading control for Figure 11A.
