# Supplementary material for: Fecal microbiota transplantation alleviates DSS-induced colitis: increased fecal butyrate, reduced colonic p65 phosphorylation, and altered Th17/Treg ratios in the spleen and mesenteric lymph nodes
Source: Front Immunol. 2026 Aug 17;17:1903198. doi: 10.3389/fimmu.2026.1903198 (PMC13527040; doi:10.3389/fimmu.2026.1903198)
Supplement: Supplementary file 2 [file Table2.docx]

**Supplementary Figure S1 | Gating strategy for flow-cytometric identification of Th17 and Treg cells in the fecal-microbiota-transplantation (FMT) study.**

Representative bivariate density plots show the sequential gating hierarchy, applied identically to both tissues and to all groups of the FMT experiment (Normal, DSS, FMT and 5-ASA; quantified in Fig. 5), illustrated for a representative normal-control (NC) sample. **Top row (a–e)**, mesenteric lymph node; **bottom row (f–j)**, spleen. **(a,f)** Lymphocytes were gated on forward- versus side-scatter area (FSC-A/SSC-A). **(b,g)** Single cells were selected on FSC-A versus FSC-H to exclude doublets. **(c,h)** CD4^+^ T cells were identified within single lymphocytes. **(d,i)** Regulatory T (Treg) cells were defined as CD4^+^CD25^+^Foxp3^+^ and **(e,j)** T-helper-17 (Th17) cells as CD4^+^IL-17A^+^ cells, both gated on the CD4^+^ population defined in (c) and (h), respectively. Numbers adjacent to each gate denote the percentage of events within the parent gate. Prior to staining, cells were stimulated with PMA and ionomycin in the presence of brefeldin A, Fc-blocked (anti-CD16/CD32), surface-stained for CD4 and CD25, then fixed, permeabilized and intracellularly stained for Foxp3 and IL-17A. Samples were acquired on a Raise Cyte flow cytometer (Qingdao Raise Care Biotechnology Co., Ltd.) using FITC/PE/APC/PE-Cyanine7 detectors with single-stain compensation, and analysed in FlowJo v7.6.1. The upstream scatter, singlet and CD4^+^ gates (a–c, f–h) were reconstructed from the raw .fcs files of the representative samples, whereas the terminal Treg and Th17 plots (d,e and i,j) are the corresponding compensated exports from the acquisition software (parent gate, CD4^+^; event count indicated on each plot).

**Antibody panel (identical for both experiments).**

| **Target** | **Fluorophore** | **Clone** | **Supplier (cat. no.)** |
| --- | --- | --- | --- |
| CD4 | PE | GK1.5 | Elabscience (E-AB-F1097D) |
| CD25 | PE-Cyanine7 | PC-61.5.3 | Elabscience (E-AB-F1102H) |
| Foxp3 | FITC | FJK-16s | Elabscience (E-AB-F1351C) |
| IL-17A | APC | TC11-18H10.1 | Elabscience (E-AB-F1199E) |

**Supplementary Figure S2 | Gating strategy for flow-cytometric identification of Th17 and Treg cells in the sodium-butyrate (NaB) / NF-κB-inhibitor (PDTC) study.**

Representative bivariate density plots show the identical sequential gating hierarchy applied in the second experiment (Control, DSS, NaB, PDTC and PDTC+NaB; quantified in Fig. 12), illustrated for a representative control sample. **Top row (a–e)**, mesenteric lymph node; **bottom row (f–j)**, spleen. **(a,f)** Lymphocytes were gated on forward- versus side-scatter area (FSC-A/SSC-A). **(b,g)** Single cells were selected on FSC-A versus FSC-H to exclude doublets. **(c,h)** CD4^+^ T cells were identified within single lymphocytes. **(d,i)** Regulatory T (Treg) cells were defined as CD4^+^CD25^+^Foxp3^+^ and **(e,j)** T-helper-17 (Th17) cells as CD4^+^IL-17A^+^ cells, both gated on the CD4^+^ population defined in (c) and (h), respectively. Numbers adjacent to each gate denote the percentage of events within the parent gate. Prior to staining, cells were stimulated with PMA and ionomycin in the presence of brefeldin A, Fc-blocked (anti-CD16/CD32), surface-stained for CD4 and CD25, then fixed, permeabilized and intracellularly stained for Foxp3 and IL-17A. Samples were acquired on a Raise Cyte flow cytometer (Qingdao Raise Care Biotechnology Co., Ltd.) using FITC/PE/APC/PE-Cyanine7 detectors with single-stain compensation, and analysed in FlowJo v7.6.1. The upstream scatter, singlet and CD4^+^ gates (a–c, f–h) were reconstructed from the raw .fcs files of the representative samples, whereas the terminal Treg and Th17 plots (d,e and i,j) are the corresponding compensated exports from the acquisition software (parent gate, CD4^+^; event count indicated on each plot). The antibody panel, instrument, compensation and analysis software were identical to those in Supplementary Fig. S1 (antibody panel above).
